# How to fix a broken heart—designing biofunctional cues for effective, environmentally-friendly cardiac tissue engineering

**DOI:** 10.3389/fchem.2023.1267018

**Published:** 2023-10-12

**Authors:** Aleksandra Benko, Thomas J. Webster

**Affiliations:** ^1^ AGH University of Science and Technology, Krakow, Poland; ^2^ Department of Biomedical Engineering, Hebei University of Technology, Tianjin, China; ^3^ School of Engineering, Saveetha University, Chennai, India; ^4^ Program in Materials Science, UFPI, Teresina, Brazil

**Keywords:** CVD (cardio vascular disease), cardiac tissue bioengineering, mechanostimulation, electrostimulation *in vitro*, bioactivity, biofuctionality, green materials, green design

## Abstract

Cardiovascular diseases bear strong socioeconomic and ecological impact on the worldwide healthcare system. A large consumption of goods, use of polymer-based cardiovascular biomaterials, and long hospitalization times add up to an extensive carbon footprint on the environment often turning out to be ineffective at healing such cardiovascular diseases. On the other hand, cardiac cell toxicity is among the most severe but common side effect of drugs used to treat numerous diseases from COVID-19 to diabetes, often resulting in the withdrawal of such pharmaceuticals from the market. Currently, most patients that have suffered from cardiovascular disease will never fully recover. All of these factors further contribute to the extensive negative toll pharmaceutical, biotechnological, and biomedical companies have on the environment. Hence, there is a dire need to develop new environmentally-friendly strategies that on the one hand would promise cardiac tissue regeneration after damage and on the other hand would offer solutions for the fast screening of drugs to ensure that they do not cause cardiovascular toxicity. Importantly, both require one thing–a mature, functioning cardiac tissue that can be fabricated in a fast, reliable, and repeatable manner from environmentally friendly biomaterials in the lab. This is not an easy task to complete as numerous approaches have been undertaken, separately and combined, to achieve it. This review gathers such strategies and provides insights into which succeed or fail and what is needed for the field of environmentally-friendly cardiac tissue engineering to prosper.

## 1 Introduction

Cardiovascular diseases (CVDs) are some of the most common death-causing diseases, culminating into roughly 18 million deaths per year. 85% of these cases are due to stroke or myocardial infraction (World Health Organization, 2017). These conditions almost always require lengthy hospitalization stays, which is connected to the significant use of energy and resources, meaning that the healing process itself has a significant economic and ecological impact. As just one of many CVD examples, myocardial infraction survivors will suffer from mild to severe heart tissue damage within a few hours after the infraction and insufficient blood supply may kill up to 25% of cardiomyocytes with normal cardiac tissue irreversibly replaced by scar tissue because of the heart’s low tissue regenerative potential (Laflamme and Murry, 2011; Maher, 2013). This leads to changes in the heart’s mechanical properties–the tissue becomes stiffer and so the muscles need to perform more work to produce the same contraction, leading to reduced efficiency of this vital organ (Laflamme and Murry, 2011; Tzahor and Poss, 2017). As a result, a large share of patients who have suffered from myocardial infraction may never go back to functioning the way they used to before the incident. The overall deterioration of life quality is not the only downside of CVDs as such a state of matters also poses a large socioeconomic issue. Many patients will be permanently placed on drugs or have some sort of medical device implanted (such as pacemaker or stent derived from non-environmentally friendly materials), further increasing the environmental carbon footprint resulting from CVDs. Hence, although not discussed frequently enough, there is a large amount of socioeconomic and ecological pressure to design environmentally-friendly strategies for healthy cardiac tissue regeneration.

The negative environmental impact of developing and using biomaterials (including those for cardiac tissue regeneration) cannot be understated. For example, none of the polymers currently FDA approved as biomaterials are considered environmentally-friendly and all leave a large carbon footprint either during manufacturing or use. Further, with only 16% of all polymers recycled today, there is a growing concern over greenhouse gases emitted during polymer synthesis which is expected to increase from 850 metric tons in 2019 to 2.8 gigatons in 2050. Apart from this, there is also a dire need to develop fast and reliable screening strategies that could fast track drug approval and treatment strategies from the lab into the market, while reducing the environmental impact that comes from laboratory animal testing, *in vitro* screening, and associated costs. It is worth noting that toxicity towards the heart is one of the most serious side effects of numerus drugs from fighting COVID-19 to diabetes, which attributes to roughly 20% of pharmaceuticals being withdrawn from the market (Siramshetty et al., 2015; Onakpoya et al., 2016). Collectively, all of these issues point to a significant environmental crisis with current CVD research and products. To date, many researchers have focused on saving human health through the design of improved CVD biomaterials but have omitted the loss of human life that results from environmental damage.

**FIGURE 1:**
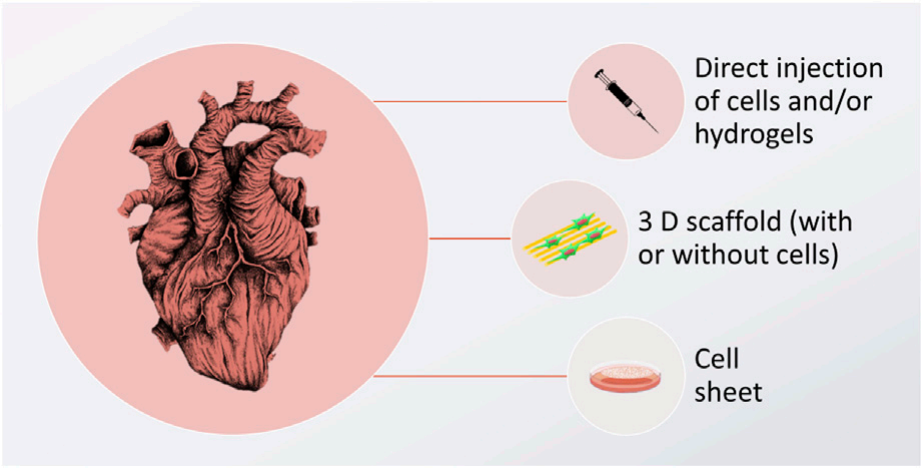
Graphic depiction of cardiomyocyte delivery approaches, aimed to induce cardiac tissue regeneration. The image of the heart is adapted from “ink heart” by Julia Bramer, CC BY-NC 4.0, graphical scheme designed by TinyPPT.com and the cell sheet is reprinted from Yamato et al. (2007) with permission form Elsevier, Copyright 2007.

Critical to future CVD biomaterial solutions involve developing green biomaterials as well as the use of cells. Because there is no stem cell niche in the heart that would allow recruitment of new cardiomyocytes (Karbassi et al., 2020), cardiac tissue does not self-regenerate (Laflamme and Murry, 2011). Various tissue-engineering strategies have been suggested to foster heart healing, and some of the most common assume that new cardiomyocytes (or their progenitors) need to be delivered to a scarred tissue site, resulting in its remuscularization. In this approach, it is envisioned that the newly delivered cells will become integrated into the target tissue, followed by new tissue formation, thus inducing the remuscularization process. Cells can be delivered by direct injection, inside the cell sheets, or growing within the 3D scaffolds. These 3 approaches are graphically depicted in Figure 1.

While direct injection of cells is the simplest approach with a low impact on the environment, it has little chance of encouraging the cells to stay at the desired diseased place, resulting in low cellular retention (<1%), which often causes this strategy to be ineffective. Cell injections have also been reported to cause arrythmia (Zimmermann, 2020) and they do not come with the support from other cells, such as cardiac fibroblasts, which are known to play a crucial part in guaranteeing proper cardiomyocyte function (Fujita and Zimmermann, 2017; Zimmermann, 2020). One way to circumvent this is injecting cells with supportive gel-forming biomaterials. These biomaterials are liquid when mixed with cells and gel upon delivery to the target area. While this strategy forces the cells to stay in the designated damaged area of the heart, it is limited to treating small defects only; as such implantable biomaterials do not have sufficient mechanical strength, or complex architecture to temporarily substitute for the function of the damaged tissue.

Another approach is to fabricate a cell sheet construct–a cellular co-culture growing in an extra-cellular matrix (ECM) synthesized by the cells themselves (Yamato et al., 2007). Here, the main limitation lies in the fact that these constructs are hard to handle, and their implantation may be troublesome, especially when large defects are to be treated. It is also difficult to simulate the mechanical, physicochemical, and electrical properties of the heart itself in such constructs.

A third option involves using a scaffold that is tailored to mimic the native ECM, while still providing a satisfactory shape and size stability. Preferably, such scaffolds would be made of “green materials”, i.e., derived from natural, renewable sources, such as plants or animal tissues, synthesized by microorganisms (bacteria or fungi), or reconstituted from different industry by-products (such as the food industry). On such scaffolds, various cell types can be seeded concurrently. Additionally, various biofunctional cues can be introduced to enhance cellular proliferation and differentiation into cardiomyocytes. In this approach, cells are cultured on the scaffold for a given amount of time, until they reach the desired differentiation and maturation state, at which point epicardial implantation can be performed. Such 3D scaffolds are favorable in that they may not only be used for the creation of CVD implants, but they can also serve as *in vitro* drug screening platforms in the lab. By using such artificial tissue constructs, the length and cost of new drug development can be reduced. At the same time, the extensive environmental impact of conducting traditional *in vitro* and *in vivo* studies can be mitigated.

It is generally acknowledged that these man-made constructs should be able to replicate the cellular natural environment as closely as possible. Then, the cells behave as they would within the natural healthy heart, reconstituting healthy native tissue. Hence, the scaffold should bear characteristics similar to functional tissues. For this reason, a set of physicochemical properties of the healthy myocardium is presented in Table 1.

**TABLE 1:** Chemical composition and basic physicochemical properties of the native healthy cardiac tissue.

Structural compounds	Other compounds	E-modulus	Contraction force	Electrical conductivity	Conduction velocity	Excitability	ECM morphology	Type and number of cells
Laminin; Type I, III and IV collagen; elastin; fibronectin Sharma et al. (2019)	Proteoglycans, glycosaminoglycans, etc. Sharma et al. (2019)	10–50 kPa Engler et al. (2008); Chopra et al. (2012); Zimmermann (2020)	0.2–50 mN/mm^2^ Zimmermann (2020)	0.06–0.4 S/m Miklavčič et al. (2006)	10–50 cm/s Zimmermann (2020)	1–3 Hz under electrical stimulation Zimmermann (2020)	Aligned, hierarchically organized fibers Kim et al. (2010); Zhang et al. (2010)	Around 10 billion cells, ¼ of which are cardiomyocytes Tirziu et al. (2010)

This review will provide some necessary background information needed to understand the subject, but the main focus will be the evaluation of some recent cardiac tissue engineering publications with special emphasis placed on using biopolymers and green-derived biomaterials, combined with specifically designed biofunctional cues. The most advanced strategies that can be combined to obtain multifunctional, biomimetic scaffolds are gathered, providing valid information about recent trends and directions into which the field is evolving to both save human health and the environment.

## 2 Cell sources

**FIGURE 2:**
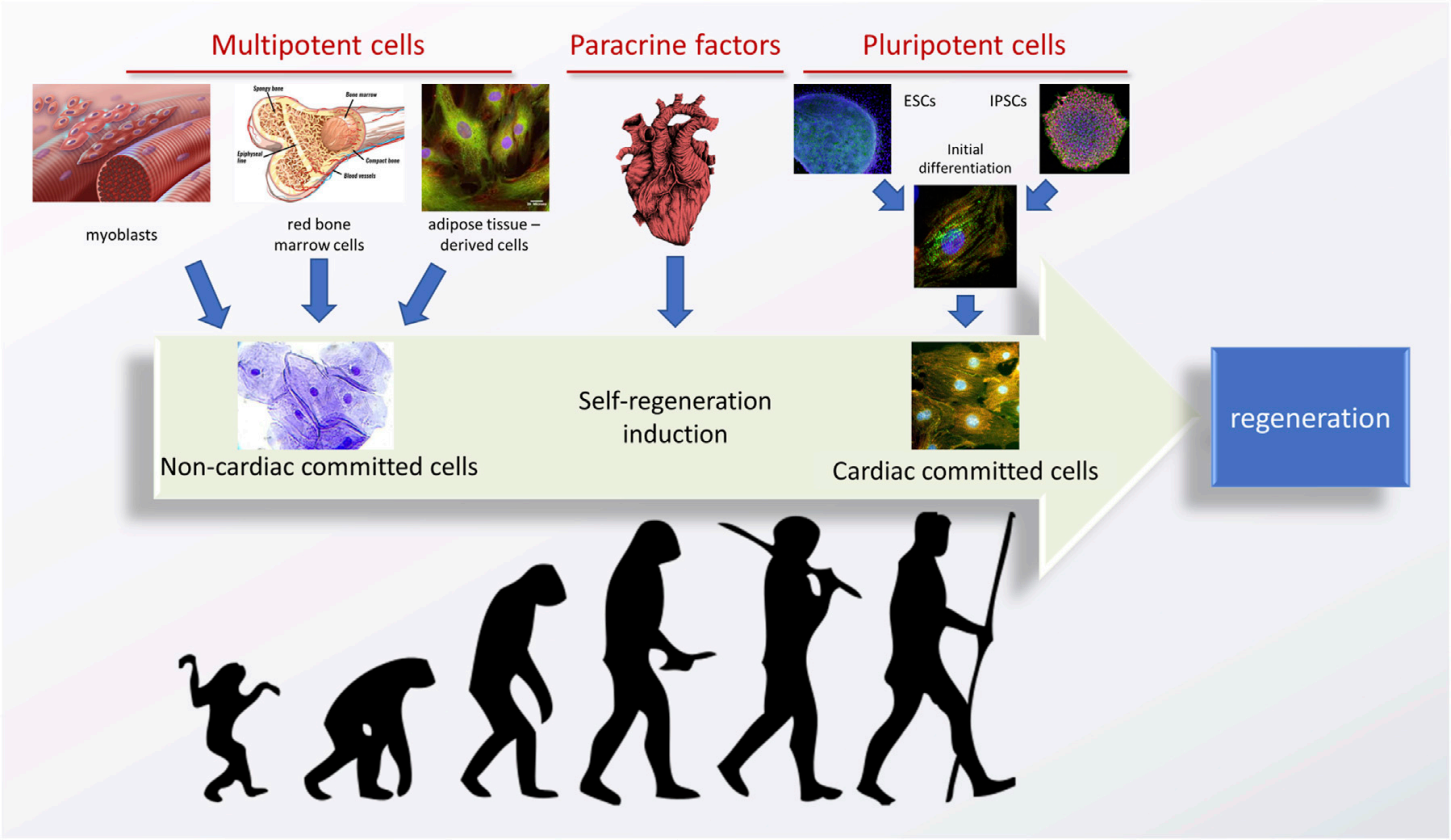
An evolution of stem cell-based strategies for tissue engineering of the heart. Adapted from (Silvestre and Menasché, 2015), using the images and graphs reproduced from an open source library—Flickr.com. Image of the heart is adapted from “ink heart” by Julia Bramer. All is reprinted under the CC BY-NC-ND 4.0 license.

A successful green cardiac tissue engineering strategy consists of two, equally important components: a properly selected, biofunctional and biomimetic environmentally-friendly scaffold and an accurate cell source. Additionally, exogenous stimuli can be employed. Because the optimal goal is to obtain cardiomyocytes of an adult phenotype, preferably autologous, stem cells are the go-to candidates. A graph representing an evolution of cardiac tissue regeneration strategies from the cells’ perspective is given in Figure 2.

Historically, the first attempts of inducing cardiac tissue regeneration revolved around multipotent (or progenitor) cells, obtained from the stem cell niches of an adult organism. These most often included bone marrow or muscle (satellite) cells, but can also include adipose tissue. While multipotent cells have a relatively high differentiation potential, they can only differentiate into closely related cells, from the same tissue type, and their proliferation potency is also lower than that of the pluripotent cells. Such therapies were able to induce a certain therapeutic effect–for example, the cells derived from bone marrow could differentiate into fibroblasts, which are cardiomyocyte supporting cells in the heart, leading to an improvement of some of the heart functions. Still, this effect was not permanent and complete regeneration of the heart was elusive because no new cardiomyocytes could be formed or recruited.

An obvious alternative was to use multipotent cardiac stem cells, obtained from the cardiac niche. In two different approaches, either the niche was identified based on cell harvesting and *in vitro* culturing, or paracrine factors were delivered into the heart with an aim to activate the already existing cells for *in vivo* regeneration. Despite the obvious logic behind this reasoning, good therapeutic effects were never obtained, and the cardiac stem cell niche was never found. Currently, it is believed that the cardiac stem cell niche is non-existent (Karbassi et al., 2020), with an extremely low regenerative potential of the heart being an obvious proof-of-concept.

A third possible source of cardiac cells are pluripotent cells. These can be obtained either from embryos (ESCs, which raises ethical concerns and is a very limited source) or induced by genetic reprogramming from somatic cells–these are called induced pluripotent stem cells (iPSCs) (Silvestre and Menasché, 2015; Zimmermann, 2020). Important advantages of using iPSCs over other cell sources are their unlimited source and ability to obtain patient-specific cardiomyocytes. The latter becomes favorable when considering tissue engineering strategies with a reduced rejection risk, as well as designing strategies for the *in vitro* evaluation of various genetic diseases (Martyniak et al., 2023). Pluripotent cells can be subjected to initial differentiation, which already has been proven to yield cardiomyocytes *in vitro*. However, these cells possess a juvenile phenotype, not able to perform as the adult cells would. Thus, their practical application is still elusive and efficient strategies to boost the cells’ maturation are needed. As various studies have proven, certain stimuli can aid this effect. One of the approaches suggests aggregating cells into specific organoids (Mills et al., 2019), but it has not been proven that this strategy significantly speeds up their maturation. Still, some increase in cardiomyocyte functionality has been reported when these cells are co-cultured with other cells (Varzideh et al., 2019).

Some very recent and promising approaches used to enhance the maturation of iPSCs-derived cardiomyocytes (iPSCs-CMs) are gathered in a review by Martyniak et al. (Martyniak et al., 2023). This excellent study provides a detailed description of the natural cardio genesis, lists important hallmarks of mature cardiomyocytes, as wells as gives validated protocols enhancing cell maturation efficacy *in vitro.* The readers are kindly referred to this review for a more extensive evaluation of various biochemical, electrical, and mechanical stimulants useful for promoting cardiomyocyte function. Instead, this article will focus mostly on strategies employed for the design, synthesis, and characterization of environmentally-friendly CVD biomaterials. Biomaterials serve as a critical support for cellular adhesion forcing the cells into specific desirable shape. Specific biomaterial morphology, chemical composition, and alignment can all enhance cellular maturation. These effects can be further boosted by selecting proper mechanical, electrical or biological cues. In the following sections, different types of available cues and/or biofunctional properties are listed, together with some exemplary cardiac tissue regeneration results, as reported in recent studies.

## 3 Co-cultures

Another important aspect of growing stem cells (such as derived CMs) *in vitro* is to understand what happens naturally during cardiac tissue development *in vivo.* The heart is composed of multiple cell types, fibroblasts being the second most abundant. Their main role is remodeling of the ECM and secreting signaling molecules that alter CM metabolism–from proliferation through maturation and growth (hypertrophy) (Li et al., 2017a; Karbassi et al., 2020; Martyniak et al., 2023). This clearly indicates that it might be very difficult, if not impossible, to obtain CMs of a mature phenotype in the absence of fibroblasts. But conducting such *in vitro* cultures is troublesome as fibroblasts proliferate much faster than CMs and can easily overtake a CM culture. Hence, different strategies have been formed to isolate the cells from one another, while still allowing the cells to exchange extracellular molecules. Some examples wherein two cell types are cultured in transwell inserts or where media is exchanged between the two separate cultures are reported throughout the literature, yielding positive outcomes (several examples are gathered in Table 2). Another possibility that has been reported in the literature is to fabricate a specific culture chamber/device, most often based on microfluidics, wherein two or more cell types are grown in separate, semi-permeable chambers, allowing for an exchange of nutrients. An example of such a solution can be found in a study by Veldhuizen et al., wherein a specifically designed co-culture yielded cells with improved cell maturation markers (Veldhuizen et al., 2020). A similar approach was also undertaken by Ronaldson-Bouchard (Ronaldson-Bouchard et al., 2018). These studies are further analyzed in Section 4 of this article.

**TABLE 2:** Some strategies to conduct cardiomyocyte—fibroblast co-cultures and their positive outcomes.

Type of culture	Administration route	Cell type	Studies conducted	Results	Ref
• Mice ESC-CM cultured with endothelial cells • hESC-CMs and mice ESC-CMs transfected with four miRNAs: miR-125b-5p, miR-199a-5p, miR-221, and miR-222	Transfected with miRNAs using Pepmute as a transfection agent	Embryonic-stem cell-derived cardiomyocytes (human and mouse), cultured with fibroblasts or endothelial cells	Immuno—fluorescence staining for α-sarcomeric actinin (α- actinin; red) and DAPI Patch-clamp Western blot TEM	• Each miRNA in the miR-combo can target ErbB4 • Knockdown of ErbB4, alters aspects of ESC-CM morphology, gene expression, and protein expression, in accordance with a more mature phenotype • Cocultures with endothelial cells: • mESC-CMs had elongated cell shape and improved alignment • Increased ratio of α-/β-MHC (myosin heavy chains isomorphs) • Properly aligned Z bands • Faster Ca^2+^ decay times • miRNA • Aligned, mature myofibrils in the cytoplasm • Significant enhancement of the α-/β-MHC ratio • More organized sarcomeric structures, I bands, and well-formed cristae • Faster Ca^2+^ decay times • Increased protein level of the cardiomyocyte gap junction component Cx-43 • hESC-CMs—transfected with the same miRNA, with similar results, but also: • More negative resting membrane potential and a larger amplitude of action potential • Lower expression of ANF and higher expression of Cx-43 and KIR2.1 • After 2 months: a more organized distribution of Cx-43 and enlarged cell size	Lee et al. (2015)
• Co-culture of iPSC-CMs and hMSCs, • iPSC-CMs and hMSCs cultured using transwell inserts A cocktail of: VEGF, bFGF, SDF-1, GM-CSF, exosomes with sphingolipids and RNA Concentration is not defined	Co-culture for 3 days after differentiation	iPSC-CMs + hMSCs	cTnT-positive cell proportion qRT-PCR of cardiac markers Western blot Immunostaining TEM analysis Motion analysis Intracellular calcium ratiometric dye fluo-8 assay Oxygen consumption rate	• hMSC produce: VEGF, bFGF, SDF-1, GM-CSF (highest OCR), exosomes with sphingolipids and RNA impacted mRNA secretion, highest increase of motility • CM+SF: • Higher % of cTnT positive cells • Increased MYH7 expression Increased (MHC-β)-to-MHC-α ratio • Increased sarcomeres length, observable H-bands • Myofibrils with immature A- and I-bands, mitochondria with distinct cristae, and gap junctions, and intercalated disks at the intercellular junction • Increased beating area, acceleration, contraction, and relaxation velocity • 2.5 Hz pacing, lower beating rates, higher peak ration, longer peak duration • Higher basal respiration, spare respiratory capacity, OCR metabolic potential, and ATP production • Higher immunity towards ROS due to higher levels of STC-1 • Higher expression of mRNA1, mRNA133, mRNA208, and mRNA499 • CM+MSC • Sarcomeres with H-bands • Aligned myofibrils with clear A- and I-bands, mitochondria with more distinct cristae, and a high density of intercalated disks to which actin filaments attached • Increased contractility • Higher expression of mRNA1, mRNA133, mRNA208, and mRNA499	Yoshida et al. (2018)

## 4 Biofunctional cues

**FIGURE 3:**
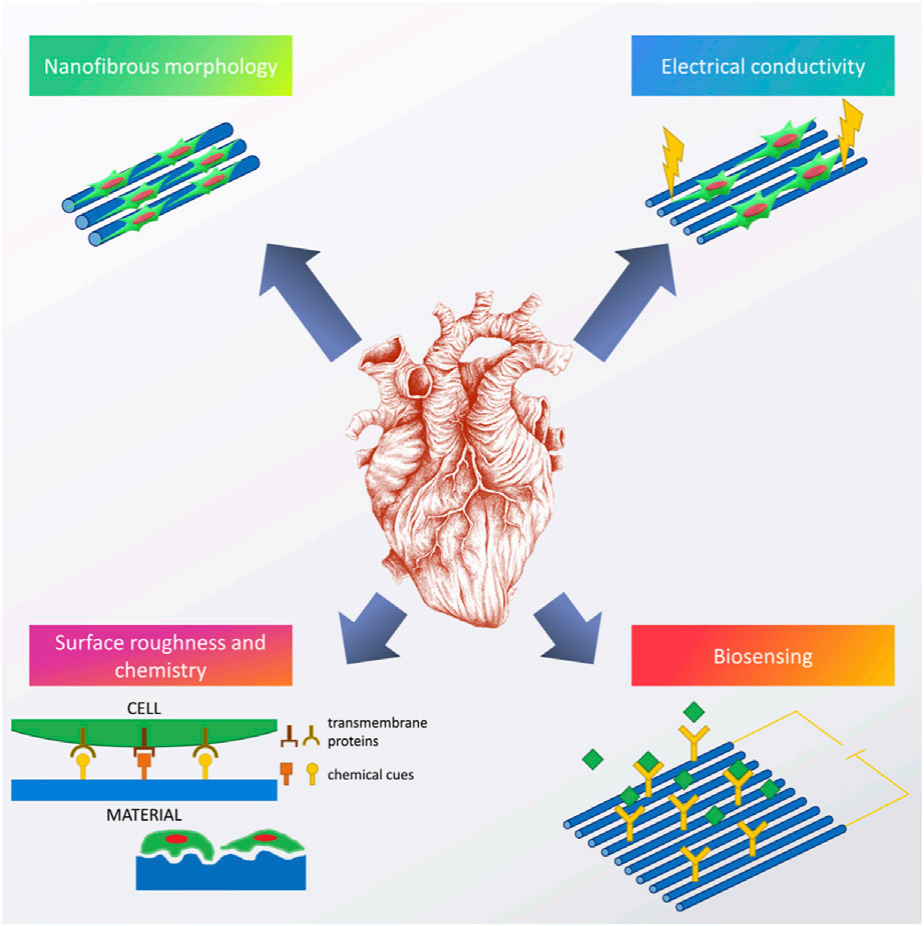
Examples of biofunctional cues used to enhance the performance of cardiac scaffolds. Image of the heart is adapted from “ink heart” by Julia Bramer, CC BY-NC 4.0.

Constant progress in the field of biomaterials and other overlapping sciences (i.e., biology, chemistry, materials science, etc.) has led to an understanding that efficient regeneration of tissues cannot be completed without introducing proper biofunctional cues (Ratner et al., 2013a). For cardiac tissue engineering, examples of the most commonly reported biofunctional cues can be found in Figure 3.

Most of the current cardiac tissue engineering strategies are based on the idea that reproducing morphology and functionality of the natural cardiac ECM is the best way to guarantee successful regeneration (Dvir et al., 2010). As gathered in our recent chapter, cardiac ECM is mostly fibrous (Benko et al., 2022) and reproducing its morphology roots back to 2004, with recent articles proving a facility of doing so (Kitsara et al., 2017). However, the tricky part is to recreate not only the specific morphology, but also to mimic the chemical composition favorably, and include some additional functions.

Right now, it seems that the majority of scientific efforts in this field are focused on enhancing the as-obtained scaffolds with additional functions, thus creating highly biofunctional, smart biomaterials that would fit well into the category of third-generation biomaterials (Ratner et al., 2013a). Among others, fabricating materials with biomimetic mechanical properties (Young’s modulus of 10–50 kPa) and electrical conductivity (between 0.06 and 0.4 S/m (Miklavčič et al., 2006)), seems to be the most important. Thus, such scaffolds would enable cell to cell cross-talk, electrical stimulation *in vitro,* as well as the possibility of biosensing monitoring tissue regeneration and identifying any possible pathologies occurring at the implantation site. Furthermore, introducing specific roughness and especially nanoroughness, combined with proper chemical cues, enabling programmed cellular behavior, also seem to be very interesting research avenues. In the following chapters, a review on what has been done in these fields will be given.

## 5 Biomimetic composition

When designing biomaterials for tissue engineering, the first consideration is choosing the right material for the matrix. From the mechanical point of view, soft tissues like cardiac tissue are the most biomimetic to polymers than to any other material group. Among these, the easiest ones to work with are synthetic polymers, like polylactide (polylactic acid, PLA), polyglycolide (polyglycolic acid, PGA), polycaprolactone (PCL), polyurethanes (PU), or polyglycerol sebacate (PGS). Synthetic polymers have relatively good processability, i.e., can easily be tailored into specific shapes using standard and novel materials processing techniques such as: molding, various 3D printing techniques, freeze drying, and different spinning techniques. Apart from facile processing, some synthetic polymers are already approved by regulatory agencies for medical use as they guarantee high repeatability of the final product, and so, are relatively cost-effective to obtain a high-quality final product with desired properties. For this reason, these materials have dominated the field and there already have been some extensive reviews about their use in cardiac tissue engineering (McMahan et al., 2020; Trombino et al., 2021).

**FIGURE 4:**
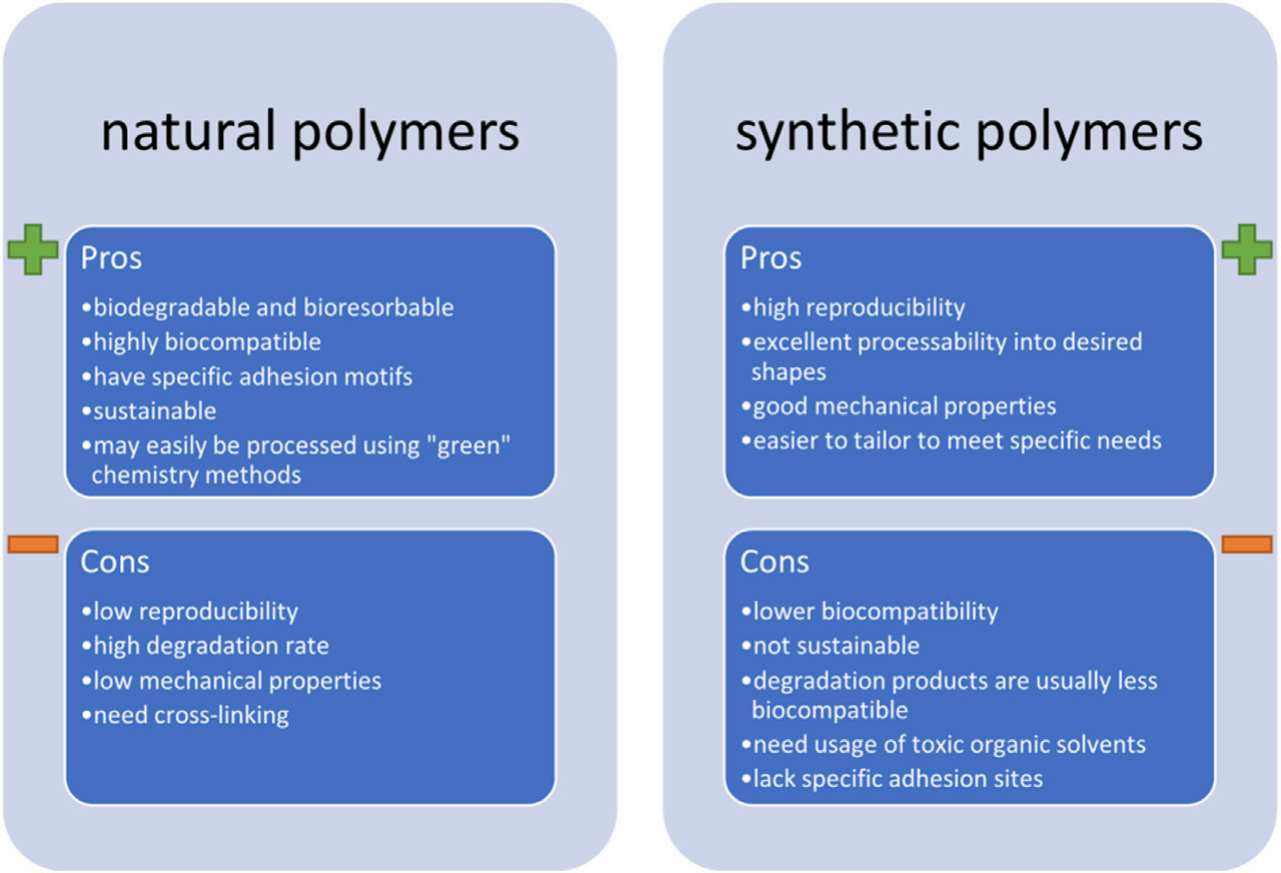
The most important pros and cons of using natural and synthetic biopolymers in fabricating scaffolds for cardiac tissue engineering.

Still, it is important to keep in mind that the use of synthetic polymers has some important disadvantages. First off, there is low chemical biomimetism, reducing the chance of a positive reaction from cardiomyocytes, such as improved cell maturation or differentiation. Next, while some of them might be biodegradable (meaning they decompose into products that are safe for the environment and the living organisms), they are rarely bioresorbable (meaning that their products are incorporated into the natural metabolic cycle of cells). Further, these materials are far from green, i.e., they are rarely obtained from sustainable sources and their processing requires usage of toxic solvents (such as cholorform). All of these factors contribute to a relatively high environmental carbon footprint of synthetic biomaterials. A comparison of some of the most important pros and cons of synthetic and natural polymers are gathered in Figure 4.

All of the above-mentioned disadvantages of using synthetic polymers contribute to the rising trend of choosing those of natural origins. These can be grouped into three main categories: plant-derived materials, cell-produced materials, and animal-derived materials. Some recent examples of using natural polymers in the field of cardiac tissue engineering are gathered in our recent book chapter (Benko et al., 2022) and the readers are kindly referred to it if more details on the subject are sought. Instead, herein we will summarize some applications which we believe are the most promising. In fact, based on the cardiac ECM composition, it is our strong opinion that modern cardiac tissue engineering strategies should focus on fabricating scaffolds majorly based on collagen, especially type I. Collagen is a predominant component of numerous tissues. As such, it is a by-product of food, hide and skin industries–it is a common waste product that should be further utilized. This takes time and resources and instead, collagen could be incorporated into modern tissue engineering strategies. The easiest way of processing collagen involves its denaturation into gelatin and this is typically done through subjecting it to high temperature, acidic or basic hydrolysis (Van Vlierberghe et al., 2014). The outcome product has a gelling ability, but is also soluble in water, and is deprived of some of collagen’s biofunctional properties. To overcome the solubility problem, gelatin can be subjected to further processing, which often involves methacrylation (Yue et al., 2015), yielding photocrosslinkable materials. Apart from that, crosslinking of the functional groups by using amide bond activators, such as EDC (1-ethyl-3-(3Dimethylamino propyl)carbodiimide hydrochloride) can also be performed. This idea was investigated in a recent study by Kumar et al., wherein core-shell fibers were prepared by covering the PCL core with a gelatin shell (Kumar et al., 2020). But crosslinking does not recuperate the lack of specific adhesion motifs of collagen. Even worse, it can reduce the amount of functional groups and side chains, which are available to cells and responsible for their adhesion. To improve its biomimicry, recent studies focus on the use of natural collagen, and in some cases, even decellularized cardiac ECM. The latter was the subject of a 2016 review article by Wang and Kristman (Wang and Christman, 2016), followed by a newer one by Sakina et al. (Sakina et al., 2020) and the readers are kindly referred to these reviews for detailed background information and advances on the subject.

In a more recent study by Blazeski et al. (Blazeski et al., 2019), thin slices of adult myocardium from pigs were decellularized and then repopulated with iPSC-CM cells. The cells readily populated the as-obtained scaffold, revealing high levels of self-organization and alignment. Adult phenotype markers were revealed, and the slice had a highly synchronized contractile behavior. In a different approach, a hydrogel derived from cardiac ECM was mixed with chitosan and alginate to obtain a mechanically stable material. Sadly, its real performance and applicability are hard to evaluate as the authors seeded it with human mesenchymal stem cells, for which differentiation into cardiac cells is highly doubtful (Tamimi et al., 2020).

An example of superior performance of collagen type I in the CMs cultures can be found in a study by Veldhuizen et al. (Veldhuizen et al., 2020), which is described in detail in Section 4. Another example is MXene modified collagen scaffold, described in Section 5. Some older examples of the superior performance of collagen in cardiac tissue engineering can be found in a 2019 review by Wu (Wu et al., 2019).

Other examples of biopolymers used in cardiac tissue engineering are: fibrin (Tao et al., 2017; Ronaldson-Bouchard et al., 2018; Ronaldson-Bouchard et al., 2019; Pretorius et al., 2022), fibronectin (Amano et al., 2016), and albumin (Fleischer et al., 2017). All of these proteins were used to fabricate thick and functional patches, seeded with either iPSC-CMs or neonatal cardiomyocytes. In a study by Amano et al. (Amano et al., 2016), co-cultures with fibroblasts and endothelial cells resulted in vascularized tissue with high levels of troponin T recorded. While the results are very promising, further evaluation of the maturity of cardiomyocytes should be performed. Still, this is an interesting and important approach to obtain functional tissues *in vitro* which can be used for drug screening. A self-organizing fibrin hydrogel was suggested for a similar application by Tao et al. (Tao et al., 2017). Seeded with neonatal cardiac cells, the gel manifested synchronous and spontaneous beating as early as on the fourth day of culture. After 6 days of culture, the patches revealed adult heart beat-like contraction rates, with high levels of α-Actinin and Cx43 observed. Certainly, in order to move forward with the study, a more detailed evaluation of the maturation markers should be performed.

A multifunctional approach in the fabrication of the cardiac tissue engineering scaffold can be found in a study by Fleischer et al. (Fleischer et al., 2017). In order to replicate the complex architecture of the cardiac ECM, an electrospun albumin nanofibrous membrane was patterned with a laser to create aligned microgrooves for culturing neonatal cardiomyocytes. Alternatively, the same electrospun scaffold was patterned into microtunnels with side cages for culturing endothelial cells and releasing vascular endothelial growth factor (VEGF), respectively. Furthermore, another version of the same scaffold was processed to contain dexamethasone (DEX). The free layers were assembled into a thick scaffold, glued together with an ECM. This was used for the co-culture of two cell types and controllable release of factors known to speed up the CMs’ maturation (specifically, VEGF and DEX). It was revealed that CMs align with the fabricated patterns, are elongated, have a massive striation, and show upregulation of Cx43. An oriented propagation of electrical signal was observed. Sadly, the complex 3D architecture was not evaluated *in vitro* for its ability to induce cardiomyocyte maturation. Instead, it was implanted subcutaneously into a rat’s back to reveal its ability for neovascularization. Hence, further studies should be undertaken for evaluating the applicability of this system to *in vitro* cultures.

## 6 Chemical and biological modification

**FIGURE 5:**
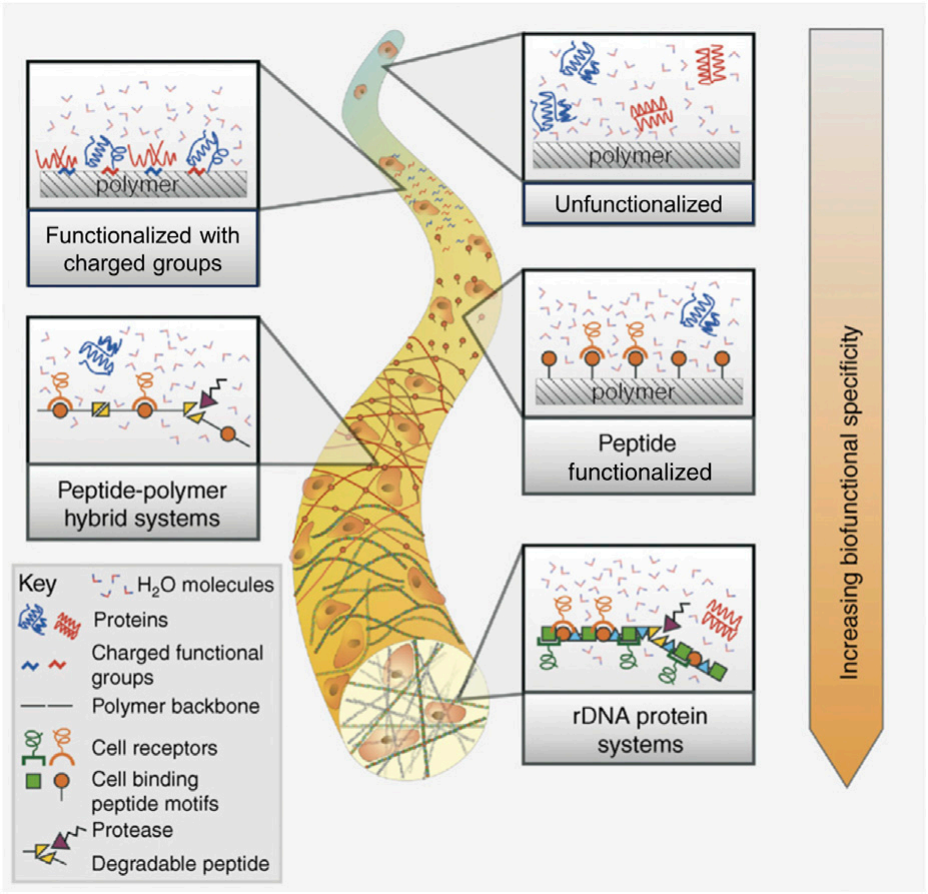
Strategies to enhance a biomaterial’s functionality by introducing chemical and biochemical cues (surface and in-mass modification). Reprinted with permission from I.C. Bonzani, J.H. George, M.M. Stevens, Novel materials for bone and cartilage regeneration, Current Opinion in Chemical Biology 10 (6) (2006) 568–575, Elsevier 2006 (Bonzani et al., 2006).

The idea of grafting the scaffolds for cardiac tissue engineering with various biological and chemical cues is often employed and as such, has been evaluated in quite a few reviews, both as a main topic (Tallawi et al., 2015; Jafarkhani et al., 2018), or as a part of a larger study (Kitsara et al., 2017). Generally, this strategy is employed to enhance the material’s functionality and thus, improve its biological performance. As with every other type of tissue engineering scaffold, this can be achieved either by surface or in-mass modifications, by physicochemical or biological methods. A graphical representation of some of the strategies used to modify a material’s functionality is found in Figure 5 (Bonzani et al., 2006). Surface modifications are most often designed to change the initial reaction of cells that come in contact with specific functional groups or peptide motifs, usually enhancing cellular adhesion, spreading and proliferation. Meanwhile, in-mass functionalization is aimed to cause more of a long-term, delayed effect, evoked by the release of active molecules: anti-inflammatory and anti-bacterial substances, specific growth factors, proteins, DNA or rDNA motifs, etc. (Tallawi et al., 2015). It is worth noting that in some cases, multi-step functionalization is justified to obtain desired results, for example, covering the material with a bioactive molecule can be preceded by surface modification via physicochemical techniques in order to introduce functional groups able to anchor the molecules (Boffito et al., 2018). In-mass and surface modifications can also be combined together to yield a material enriched with multiple biofunctional cues.

### 6.1 Physicochemical techniques

Physicochemical modification of the scaffold surface is typically performed to introduce functional groups to the surface of the material. Changing the surface free energy and altering the surface charge, can in turn have a direct effect on cell (Keselowsky et al., 2004; Thevenot et al., 2008; Nakaoka et al., 2010) and bacteria (Adolfsson et al., 2019; Duch et al., 2019) adhesion and proliferation, as well as cellular differentiation. Generally, the introduction of functional groups can be done via three alternative routes: 1) wet/dry chemistry, 2) plasma grafting/treatment, or 3) photo-induced grafting (Tallawi et al., 2015). While the first route is the cheapest, the most versatile and effective, making it possible to introduce virtually any kind of functional groups, it is also the one with the highest negative impact on the environment using aggressive, often toxic reactants. The process is hard to control and may also negatively affect the material’s bulk properties. Another disadvantage that is very troublesome is post-treatment purification, with the removal of unwanted substances requiring multiple washing steps. Still, it remains the most popular route to grant the material with a desirable type and amount of functional groups. Photo-induced grafting is safer than chemicals methods, but the availability of possible modifications is significantly slower and the efficiency of the process is relatively low. Meanwhile plasma modification, which is typically performed using oxygen or nitrogen plasma, can be used to obtain materials with a desired amount of functional groups but it is hard to control their type (for example, in O_2_ plasma, the oxidation level), and the depth of modification is typically limited to a very thin outer layer of the material, which can be insufficient for some applications (Tallawi et al., 2015; Kitsara et al., 2017; Jafarkhani et al., 2018).

In 2008, Natarajan et al. designed and performed a very important study aimed to investigate what types of functional groups are preferred by cardiomyocytes. To do so, the group performed wet chemistry modification with two types of amine-terminated chemical species ((3-aminopropyl)triethoxysilane (APTES) and trimethoxysilylpropyldiethylenetriamine (DETA)) and successive modification with self-assembled monolayers, terminated either with carboxyl or hydroxyl functional groups. It was found that the adhesion and proliferation of cells was enhanced on DETA modified surfaces, and retarded on surfaces with an abundance of hydroxyl species. Additionally, OH groups decreased the number of beating cardiomyocytes that generated longer action potentials. Thus, it had been suggested that OH groups impair cardiomyocyte viability, differentiation and maturation.

In 2014, Guex et al. modified the surface of an aligned PCL fibrous scaffold with a radio-frequency (RF) plasma process (Guex et al., 2014). The groups reported that the surface functionalization yielded biocompatible materials that supported mesenchymal stem cell adhesion. *In vivo* evaluation was completed using female Lewis rats with a surgical model of myocardial infarction and a cell-seeded and non-seeded scaffold. Non-seeded scaffolds did not alter the heart recovery process, but the cell-modified scaffold allowed for stem cell retention 4 weeks post-implantation which helped to stabilize heart function and reduce LV dilatation. The reported results are very promising and can serve as a stepping stone for further development of efficient cardiac tissue engineering scaffolds able to restore functional properties of the damaged heart. It seems that endowing the material with further biofunctional cues could yield even better results, and thus calls for further evaluation. However, it is critical to mention that PCL is not environmentally friendly, thus, this approach does harm the environment.

For more examples of surface modifications used to improve biomaterial biological performance for cardiac tissue engineering applications, the readers are kindly referred to Tallawi et al. (Tallawi et al., 2015).

### 6.2 Biological cues

**FIGURE 6:**
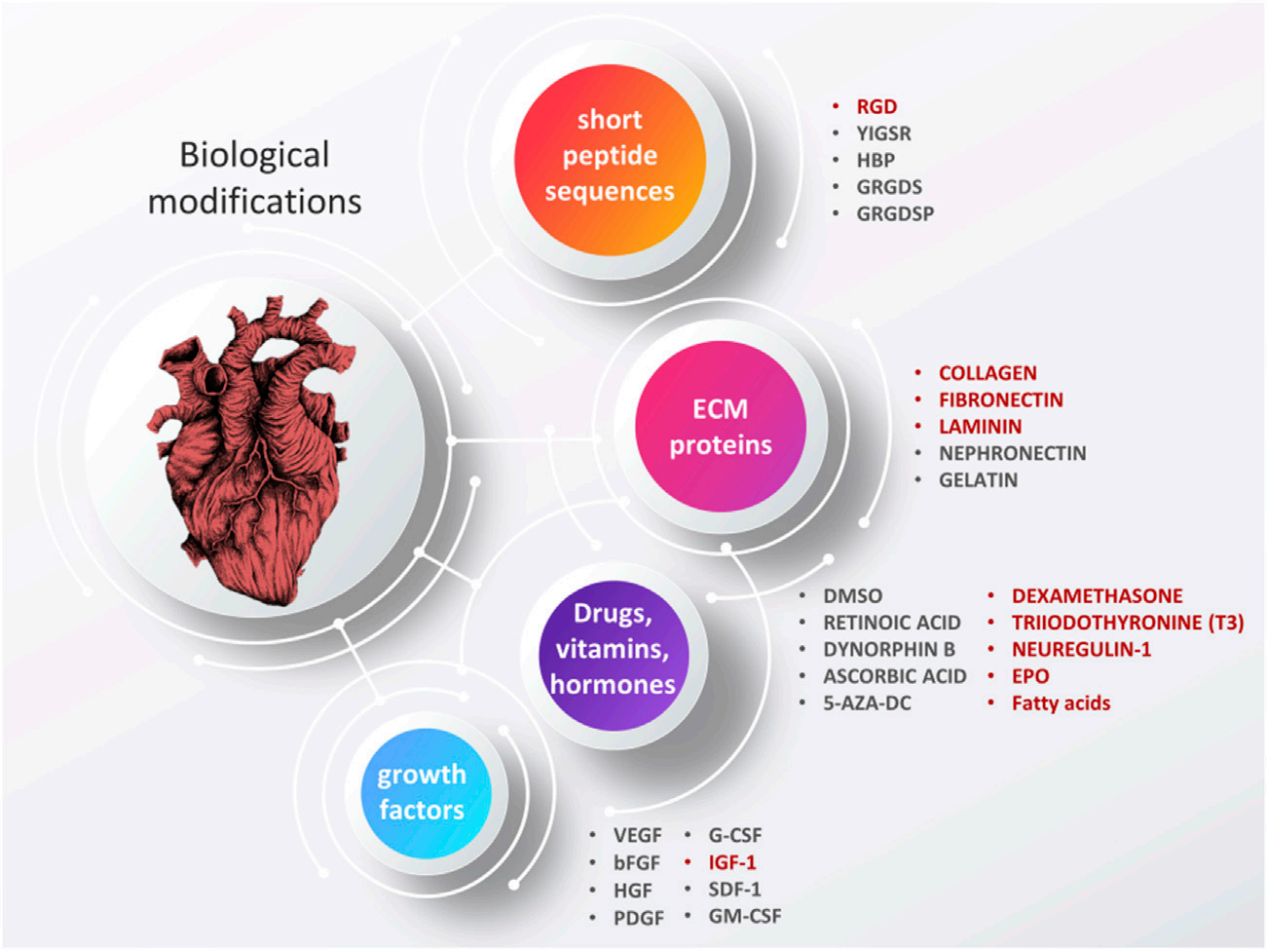
Biological modifications of cardiac tissue engineering scaffolds, aimed to grant materials with enhanced bioactive properties. Some of the additives that have shown the most promising results are highlighted in red. Based on (Tallawi et al., 2015). Image of the heart is adapted from “ink heart” by Julia Bramer, CC BY-NC 4.0, graphical scheme designed by TinyPPT.com.

As suggested by Tallawi et al., biological cues used to evoke a specific cellular reaction can be divided into four groups: short peptide sequences, ECM proteins, synthetic chemicals and growth factors (Figure 6). For a detailed description of what is used the most often and why, the readers are referred to the aforementioned article (Siramshetty et al., 2015). Here, we would like to list some of the most commonly used molecules and outline some of the more recent advances. Certainly, one of the biggest challenges in fabricating biomaterials embedded or covered with biological molecules is maintaining the biomolecule’s biological activity–this can be altered by physical and chemical interactions with polymers and dispersants or affected by processing techniques. In general, green processes are less susceptible to alter a biomolecule’s activity since they are often less aggressive, do not involved toxic chemicals, and are present in a natural form. This is most likely the main reason why recent advances in the use of more sophisticated biological modifications describe combining green materials with injectable hydrogels–in this form of administration, the risk of decreased biological activity is significantly reduced. Meanwhile, fibrous scaffolds are most often modified by covering them with natural ECM proteins and short peptide sequences, the latter giving a better chance to present receptor binding domains than the whole proteins which may become folded and denature more easily (Tallawi et al., 2015).

#### 6.2.1 ECM proteins

The ECM of the heart is built from multiple types of proteins, glycoproteins, proteoglycans, etc., but the most abundant and/or important ones are: collagen, laminin and fibronectin. At the embryonic stage of the heart, higher levels of fibronectin are responsible for cell proliferation and migration, while latter stages of heart development, growth, and functioning are associated with an increased ratio of collagen and laminin (Tallawi et al., 2015). Thus, when considering cardiac tissue engineering scaffold, it is important to plan which stage of cardiac growth one wants to simulate, and select the appropriate ECM proteins accordingly.

Fibronectin (FN) is a large molecular weight (500-kDa) glycoprotein which is known to be an ECM molecule essential in adhesion, survival and differentiation of stem cells (Kang et al., 2014). FN is secreted by mesenchymal cells and assembled into insoluble matrices which have significant biological functions in embryologic development (Limper and Roman, 1992). FN influences cellular migration and proliferation, both *in vivo* and *in vitro*, and also plays a critical role in cellular early development (Van Dijk et al., 2008). Fibronectin is organized into a fibrillar network through direct interactions with cell surface receptors (Mao and Schwarzbauer, 2005). This molecule is often used as a coating for various synthetically and naturally derived fibers to enhance cellular adhesion, spreading, growth, and proliferation (Kitsara et al., 2017; Liverani et al., 2019).

Layers of collagen can be deposited on a substrate of choice by a simple immersion technique. In cellular cultures, collagen is the main constituent of commercially available Geltrex and Matrigel (also rich in laminin) which are synthetic membrane matrices aimed to improve cellular adhesion and differentiation (Tallawi et al., 2015; Stepniewski et al., 2018). Its usage is a well-established procedure, guaranteeing good results and so, similarly, the modification of fibrous scaffolds also yields materials of improved cellular adhesion and growth. In a 2019 study by Kenar et al., collagen and hyaluronic acid were incorporated into a PLA/PCL fibrous scaffold. The collagen was harvested from human tissues, resulting in better biocompatibility and lower immune reaction. When cultured with adipose tissue-derived mesenchymal stem cells, a 3-fold adhesion increase was observed, while cultures with human umbilical vein endothelial cells revealed significantly improved vascularization. Although PLA and PCL are not environmentally friendly, if they can be avoided or a green polymer used as the scaffold, these results provide significant evidence that the obtained materials should be further studied for CVD tissue engineering applications (Kenar et al., 2019).

Laminin, which is crucial to cellular adhesion, spreading and migration is often used to modify fibrous and non-fibrous scaffolds (Tallawi et al., 2015). In 2014, Yu et al. compared the effects of modifying PLGA fibrous scaffolds with three different biomolecules: two types of short peptide sequences (RGD and YIGSR) and laminin. The RGD and YIGSR peptide sequences were added in-mass during electrospinning, while laminin was used as a covering layer. While all types of modifications improved cardiomyocyte adhesion, only laminin and YIGSR were able to enhance cellular maturation and contractile behavior. Laminin contains YIGSR motifs, so the results suggest that this short peptide sequence is essential for cardiomyocyte maturation. The authors advocate the use of YIGSR over laminin, as it can be more easily incorporated into the scaffold during fabrication with a reduced risk of denaturation, lack of necessity of additional modification steps, and higher efficiency of active molecule grafting (Yu et al., 2014). To overcome problems with the adhesion of active molecules in proper conformation, Boffito et al. subjected polyurethane scaffolds to plasma treatment, followed by laminin surface modification. Laminin modification improved cardiac primitive cell proliferation, inhibited their apoptosis and enhanced cellular maturation rate. Subcutaneous implantation in mice revealed excellent biocompatibility. Although polyurethane is not environmentally friendly, the obtained results indicated that well designed surface modification with laminin is a way to obtain highly biomimetic scaffolds, with good *in vitro* and *in vivo* performance (Boffito et al., 2018).

Other popular protein modifications include the use of nephronectin (Npnt) and gelatin. Npnt has been reported to enhance cell-to-cell communication, sarcomere maturation, alignment and synchronized contractions. Similarly, gelatin has been found to enhance cardiac cell adhesion, growth and differentiation (Tallawi et al., 2015; Jafarkhani et al., 2018). In 2011, Kai et al. had found that co-electrospinning of PCL and gelatin yielded cardiac tissue engineering scaffolds that promote cellular adhesion. When highly aligned, these fibers were able to enhance cellular attachment even furthermore and force cellular alignment–thus improving biofunctionality (Kai et al., 2011). Again, however, as with all of these studies above, the base polymer (here PCL) is not environmentally friendly leaving a large carbon footprint and should be replaced with a green polymer.

In 2017, Suhaeri et al. cultured fibroblasts on PLCL fibrous scaffolds to allow them to synthesize an ECM. After decellularization, a hybrid scaffold composed of synthetic and natural fibers was obtained. This interesting approach (although not environmentally friendly) resulted in excellent performance, with a significantly improved maturation of cardiomyocytes (Suhaeri et al., 2017).

#### 6.2.2 Short peptide sequences

Among the many different short peptide sequences, the RGD motif (Arg–Gly–Asp) is the most abundant, responsible for cellular attachment via integrin binding. As such, it is very often used in tissue engineering scaffolds, including for the heart. However, as pointed out by Yu et al., better enhancement of cellular function can be achieved using YIGSR, with results similar to laminin, but with a higher ease of use and incorporation into scaffolds (Yu et al., 2014). Other modifications may include the use of longer RGD-containing sequences which were also found to stimulate integrins that are relevant in early cardiac development (Tallawi et al., 2015).

#### 6.2.3 Growth factors (GFs)

Many types of GFs can be used to evoke specific cellular reactions, including directing stem cells along certain lineages (Sepantafar et al., 2016). A short reference of GFs used in cardiac tissue engineering is listed in Figure 6, with a description of the benefits and challenges in using those can be found in reviews by Tallawi et al. (Tallawi et al., 2015) and Hastings et al. (Hastings et al., 2015). Briefly, the most commonly employed are: VEGF (vascular endothelial growth factor), responsible for angiogenesis (and thus, survival of cells), G-CSF (granulocyte colony-stimulating factor), EPO (erythropoietin) and IGF-1 (insulin-like growth factor). G-CSF, EPS and IGF-1 have all been found to restore cardiac function, by the reduction of CM apoptosis and delay of CM ageing, respectively. In studies by Davis et al. (Davis et al., 2006) and Minato et al. (Minato et al., 2012), IGF-1 has been found to significantly enhance CM maturation. Alternatively, bFGF (basic fibroblast growth factor), HGF (hepatocyte growth factor) and PDGF (platelet-derived growth factor) have also been suggested, which are responsible for the proper growth and maturation of endothelial cells and development of an endothelium (Tallawi et al., 2015). Another growth factor recently reported to improve cardiac function *in vivo* is neuregulin-1 (NRG1). This epidermal growth factor has been found to improve cardiac function in a mouse model subjected to myocardial infraction. CM viability and proliferation were improved with increased angiogenesis. Meanwhile, fibrosis and apoptosis were significantly reduced, all indicating at least partial recovery (Chang et al., 2022). In Table 3, some examples of spectacular results obtained when using IGF-1 and NRG1 in CMs cultures are gathered, with more details regarding study methodology and outcomes.

**TABLE 3:** Some examples of positive outcomes of using growth factors in cardiomyocyte *in vitro* cultures and *in vivo* mouse models.

Growth factor used	Administration route	Cell type	Studies conducted	Results	Ref
Neuregulin-1 (50 ng per 20 mg fibrinogen)	Neuregulin-1 released from the fibrin patch (fibrinogen (20 mg/ml) thrombin (10 U/ml)	H9c2, mice model	CCK-8 assay EdU assay *In vivo*—immunofluorescence and echo	NRG-1 improves proliferation and phosphorylation of AKT *In vivo*, cardiac function is improved	Chang et al. (2022)
IGF-1—CMs suspended in 1% self-assembling peptides alone, 10 ng/ml untethered of tethered IGF-1	IGF-1 released from the self-assembled, biotinylated peptides	Neonatal cardiac myocytes from 1-day-old Sprague–Dawley pups	*In vitro*: Western blot Elisa *In vivo*: 25 ng injected into myocardium of adult rats, MI model	In tethered IGF-1 samples (supposedly sustained release):	Davis et al. (2006)
				• Phosphorylation of Akt significantly increased • Decreased slow skeletal troponin I and significantly increased cardiac troponin I • Significantly increased new protein synthesis after 14 days • Sustained release in vivo (up to 28 days, still present at 84 days). Detectable activation of Akt • Injection activates critical survival pathways and improves transplanted cell growth • IGF-1 improves the efficacy of cell therapy after infarction, preventing postinfarction ventricular systolic dysfunction as well as dilation • Significantly increased sarcomeres’ length and contraction strength	
IGF-1 released from elastin-like polypetides that were used to cover the bottom of the cell well plate 10 µg/mL (GVGVP)_67_- IGFBP4 as coating, 1 µg/mL IGFBP4 (Thy) and 1 µg/mL (GVGVP)_67_-IGFBP4 added to cultures as controls	Wnt receptor-binding domain of IGFBP4 and elastin-like polypeptides	Embryonic stem cell derived CMs	*In vitro*: Western blot, RT-PCR immunocytochemistry	• On day 24, higher expression of GATA-4 and αMHC. MF20 positive areas improved to 18% • Hanging drop EB formation culture for 5 days: IGFBP4 strongly expressed, Wnt3a expression decreased • After seeding EB on scaffold, ATA4 and MHC were highly expressed, spontaneous beating after 20 days, 65% of MF20-positive areas	Minato et al. (2012)

#### 6.2.4 Drugs

Several drugs have been reported to improve cardiomyocyte differentiation and maturation (Table 4). Among these, dexamethasone (DEX), triiodothyronine (T3) and IGF-1 seem to be the most interesting and promising. Recent articles suggest that the best positive outcomes can be obtained by combining these three (Gentillon et al., 2019; Huang et al., 2020). Different strategies suggest the use of fatty acids. In an interesting article by Yang et al. (Yang et al., 2019), fatty acids were modified with bovine serum albumin. In living organisms, albumin is responsible for binding fatty acids and transporting them inside cells. *In vitro*, unmodified fatty acids become toxic to cells as they are not able to penetrate the cell wall and instead, isolate the cells from extracellular stimulus. Hence, the hypothesis of the authors was that when fatty acids are modified with albumin, they can easily penetrate the cells. When cells recognize that their surrounding is rich in a new energy source (fatty acids instead of sugars), this might trigger alteration in their metabolism. Such alteration is characteristic of mature cardiomyocytes and hence, it was expected that this change would initiate a cascade of effects that in the end would yield a fully mature phenotype. This sound hypothesis has led to very positive outcomes after 2 weeks of culture. It can only be expected that longer cultures could guarantee successful obtainment of mature cardiomyocytes, in a relatively short period of time. More details on the positive outcomes of using different drugs are gathered in Table 4.

**TABLE 4:** Some examples of positive outcomes obtained through administration of different drugs and fatty acids into the CM cultures.

Drug type/bioactive compound	Administration route	Cell type	Studies conducted	Results	Ref
Fatty acids: palmitic, oleic, and linoleic acids (52.5 µM palmitate-BSA, 40.5 µM oleate-BSA, and 22.5 µM linoleate-BSA, 120 µM carnitine (enables fatty acids uptake)) in RPMI-B27-insulin medium	After 20 days of differentiation, fatty acids were added	iPSC-CMs	• Cells fed for 2 weeks • f-actin, α-actinin staining • Western blot • Contractile force measurement • Calcium imaging • Mitochondria functional assay • mRNA-Sequencing Analysis • qRT-PCR	• Increased cell size and sarcomere lengths, decreased circularity • Under 1 Hz electrical stimulation, significant increase in calcium peak transient amplitude, maximal upstroke and decay velocities • Improved contractile force (measured on elastomeric micropost system) • In patch-clamp experiments, increased action potential maximum upstroke velocity (by 57%), and membrane capacitance (by 24%) • Maximal mitochondrial respiratory capacity after the treatment was enhanced by 38% after fatty acid treatment • No observable increase in mRNA transcripts for myofibril genes (might be due to normalization) Long-term changes in gene expression: • Cells are shutting down de novo synthesis when external fatty acid abundant, there is an increase in transcripts for fatty acid metabolism, and a decrease in transcripts for glucose metabolism • 15 and 30 min after fatty acid treatment, AMPK Thr^172^ phosphorylation, and the level of phospho-ACC, phospho-ERK and phospho-p38 MAPK are upregulated, while phospho-Akt is downregulated • ERK and p38 transient activation are suggested to induce signal cascades that promote hPSC-CM growth and hypertrophy	Yang et al. (2019)
Dexamethasone 1 µM, 1, 2, and 3 days	DEX added to the culture medium, 27^th^ day of differentiation	hESC-CMs	• Ca^2+^ transient recordings • Force of contraction (seeding on patterned polyacrylamide gels) • Immuno-fluorescence • Electrophysiology	• Dexamethasone triggered a glucocorticoid response in hESC-CMs • No significant changes in the expression of: CACNA1C and CACNA1H, RYR2, NCX1, and SERCA2, SCN5A (Nav1.5), KCNQ1, (Kv7.1) and KCNH2 (Kv11.1), and cardiac troponin genes, phospholamban (PLN) expression was significantly upregulated • After 2 and 3 days of treatment, CaT_90_ was significantly decreased at 0.2, 0.5, and 1 Hz stimulation frequencies, with a significantly smaller time constant • NCX and SERCA functions are enhanced, contributing to the decay of the systolic Ca^2+^ transient	Kosmidis et al. (2015)
Maturation medium: • Vehicle (DMSO, 1:1000 dilution) control; • 1 μM FM (FM19G11) hypoxia-inducible factor 1α (HIF-1α inhibitor) • 100 μM WY (WY-14643) agonist of peroxisome proliferator activated receptor α (PPARα) • TDI: • triiodothyronine (T3) 100 nM • Dexamethasone 1 μM • IGF-1 100 ng/ml • 1 μM FM + 100 μM WY + TDI	*At differentiation day 4, cells aggregated into cardiac spheres, induced by activin A and BMP4:* 21 days after differentiation, maturation medium for 7 days, in the presence of 0.2mM oleic acid	iPSC-CMs	• Immunocytochemical analysis and high-content imaging analysis • Seahorse XF24 metabolic flux (OCR) • qRTPCR • MitoTracker Red flow cytometry and immunostaining • Quantification of mitochondrial DNA • Calcium imaging • Contractility assay (cells stimulated at 1Hz) • RNA-sequencing (genes in treatment group compared with pediatric left ventricle (LV, male, 6.5 months))	• In 3D cultures, increased intensity of NKX2-5, α-actinin, cardiac troponin T, and cardiac troponin I *FM + WY + TDI* • The highest levels of FAO (attributed to FM and TDI), and the highest increase in oxidation of nonfatty acid substrates (FM effect) • Significant upregulation of CD36, UCP3, FASN, KLF15, ESRRA, HADHA, HADHB, GLUT4, and PDK4 • The highest basal and maximal respiration, and ATP production, increased proton leak, non-mitochondrial respiration, and reserve capacity • Mitochondrial content is increased and 37% of cells had their mitochondria highly dispersed throughout the entire cell area • Increased maximal upstroke velocity, time to 50% peak [Ca^2+^] is reduced, with a faster Ca^2+^-transient decay, and a shorter time to 50% decay • Average maximum contraction and relaxation velocities are increased • Several biological processes are enhanced: cellular lipid metabolic process, mitochondrial respiratory chain complex assembly, organic and monocarboxylic acid metabolic processes, oxidation-reduction process, small molecule catabolic, and metabolic processes, catechol-containing compound metabolic process and lipid metabolic process (CA4, MAOA, MGLL, GPX3, PDK4, NDUFC2 and NDUFS3, CYP27A1 and CYP4F12, ADRB1) • Upregulated SLC family, voltage-gated potassium and sodium channel families • Several processes are downregulated: signaling, regulation of multicellular organismal process, extracellular structure, and matrix organization, developmental process, cell-cell signaling and adhesion, and anatomical structure development	Gentillon et al. (2019)
TDI mixture: triiodothyronine (T3) 100 nM Dexamethasone 1 μM IGF-1 100 ng/ml • Cells supplemented from 12-14 days, for 7 days *(21 days post-differentiation)* • Cells grown in monolayers *(32 days post-differentiation)* • Cells grown in 3D cardiac microtissues (CMT) *(32 days post-differentiation)*: M199, HEPES, NaHCO_3_ (5% w/v), human fibrinogen (0.75 mg/mL), and rat tail type I collagen (2.25 mg/mL) grown on microfabricated tissue gauge (μTUG) devices (image below (Legant et al., 2009; Bose et al., 2018)): 42 microwells housing a pair of flexible pillars with interpillar spacing of 500 μm, and effective spring constant of k = 0.25 μN/μm *force is measured by optically evaluating deflection of cantilevers using adobe photoshop and matlab*	12-14 days after differentiation, TDI added to the medium for 7 days Days 19–21: a) Cells incubated on glass slides: 1 day for Ca^2+^ transient recordings, 2 days for single cell confocal imaging b) *CMT generation*: 24h after seeding, TDI media added for 10 days Monolayers cultured in 6-well plates for 10 days	hiPSC-CMs, seeded at a 1.5 × 10^5^ cells/cm^2^ density on μTUG 75 (CMs) :10 (HUVECs ensure mechanical stability) :15 (FB ensure compaction) to form compact structures	• Ca^2+^ transient recordings • Single cell confocal imaging qRT-PCR • Western blot • Flow cytometry • TEM	• *In 2D*, cells had a greater length to width ratio (6.4:1), increased sizes (1170 µm^2^), with substantial sarcomeric alignment and polarized gap junction distributions. High density of elongated mitochondria adjacent to the sarcomeres is observed. Clear Sarcoplasmic Reticulum (SR) terminal cisternae were observed to be adjacent to T-tubules (dyad) that were close to the Z-lines • Phosphorylation of AKT and MTOR are increased. Protein level of PPARG, CACNA1G, ADRB1, and CHRM2 are increased • *In 3D*, cells started compacting 24h after seeding, 10 days later, the compact was 30% narrower than the control, with cells aligning along the direction of axial mechanical load, and adopting rod-shaped conformations. Longer sacromeres and intercalated disks are present, while structures resembling T-tubules are located near Z-lines. Genes related to T-tubule and Ca^2+^ homeostasis, and PPARA are upregulated Dynamic force increased steadily over the first 10 days and reached a plateau, while static force continued increasing until day 18–19, and then decreased after 25–30 days. Average magnitude of static and dynamic stress was increased. Force-frequency relation was negative in untreated, and flat in treated. Mature CMs have a positive force-frequency relation (twitch amplitude increases with increasing the stimulation frequency). The maximal speed of contraction (+dF/dt) and relaxation (-dF/dt) both increased, the total contraction duration did not change, the time from electrical stimulation to peak tension and to 50% relaxation both increased. CMTs positively responded to β-adrenergic stimulation • In both 2D and 3D • H-bands can be found • Sarcomeric genes are upregulated, MYH7 is downregulated (T3 action) Significantly increased PLN and KCNJ2 mRNA expression • Decreased MYL7 expression and increased MYL2 expression – increased population of ventricular CMs • Cells can be paced (their spontaneous beating can be overdriven) 21-24 days post-differentiation (untreated cells reach this stage 10 days later) Maximum pacing rate is increaed: 2.4 Hz in 2D, 4.0 Hz in 3D • Longer APs with prominent plateau, ADP_30_ and APD_80_ are up to 1.7 times longer Increased conduction velocity, • Ca^2+^ transients increased amplitude and had faster decay times at 50% recovery	Huang et al. (2020)

## 7 Biomimetic morphology

The ECM of the heart is fibrous, with an aligned topography (Benko et al., 2022), and some tissue engineering strategies for the heart aim to replicate that due to its importance in anisotropic mechanical, electrical, and biocompatibility properties. Further, it is documented that a typical ECM fibril diameter is between 30–100 nm (Kim et al., 2010). There are numerous literature reviews about using fibrous scaffolds, most notably, three done by: Zhao et al., in 2015 (Zhao et al., 2015), Capulli et al., in 2016 (Capulli et al., 2016) and Kitsara et al., in 2017 (Kitsara et al., 2017). In the 2017 article, M. Kitsara said that: “It is evident that more studies in the future should be performed using aligned structures which better mimic the native cardiac tissue anisotropy” and indeed, a lot more has been done on this subject from that time. In this chapter, we will focus on some important studies which have investigated the impact of aligned topography on cells.

The topography and morphology of a biomaterial surface has an important impact on cells. Size, shape, alignment, spacing, and depth of the features can all affect not only the cells’ alignment and clustering, but also, metabolism and differentiation. When the sizes of surface features are between 5 µm to tens of μm, cells are geometrically confined (a lower threshold depends on the cells’ dimensions and is bigger for larger cells) (Zinger et al., 2005). Between 70 nm and 5 μm, the sizes of the features approximate the cell’s sensorial organelles, i.e. focal complexes and focal adhesions, thus strongly contributing to cellular adhesion, morphology, migration, biosynthesis and differentiation. Below 70 nm, the size of features are smaller than the cell’s sensorial organelles and current research suggests that features smaller than 40 nm are not recognized by cells as adhesive and instead affect cells at different levels of performance (Ventre et al., 2012). When features are at the nanoscale, cells become more sensitive to their specific patterns or period of appearance. For example, in a 2010 study, Kim et al. found that when cultured on 800 nm wide grooves, separated 800 nm apart, important physiological parameters of neonatal rat ventricular CM improved, such as alignment, size, expression of the major gap junction protein, connexin 43, and directional electrophysiological properties (Kim et al., 2010). In a 2015 study by Morez et al. (Morez et al., 2015), similar grooves were found to enhance the efficiency of genetic reprogramming of iPSCs into CMs. Clearly, this bodes well for natural environmentally-friendly materials since such materials already have a natural built-in nanotexture. Synthetic polymers commonly used for CVD applications would need to be further modified for such promising nanotextures.

**FIGURE 7:**
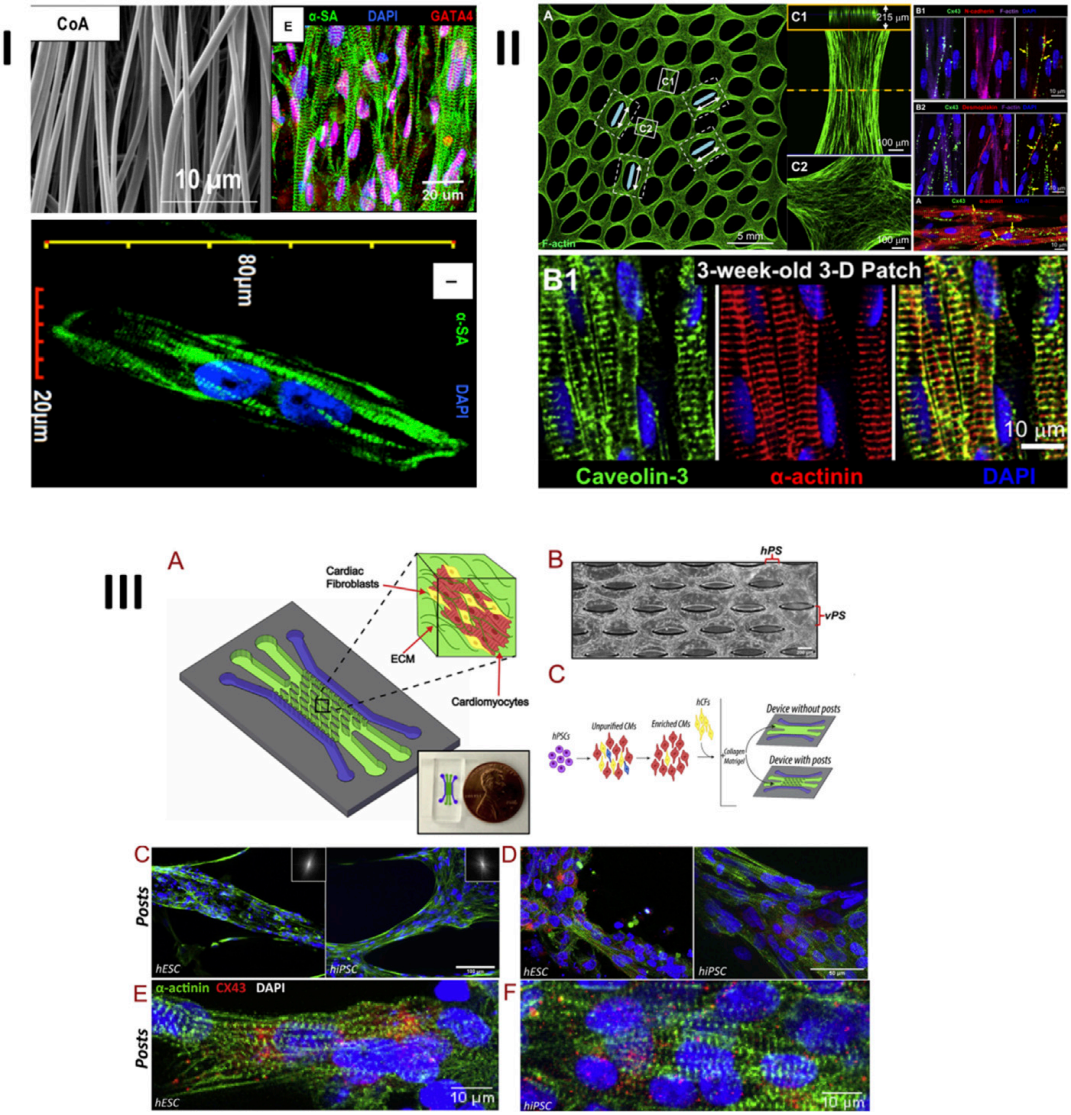
Some examples of enhanced expression of maturation markers of cardiomyocytes, cultured on different types of topographies/nanotopographies: 1) iPSCs–derived CMs cultured on electrospun aligned nanofibers; 2) neonatal rat ventricular cardiomyocytes grown on epicardial mimetics obtained by casting 10% Matrigel into biomimetic silicon molds, obtained via soft lithography, and 3) iPSCs–derived CMs and EPSc–derived CMs co-cultured with cardiac fibroblasts on a microfluidic chip. CMs are embedded inside a collagen-Matrigel hydrogel and cultured on top of microposts. I: Reprinted from Kumar et al. (2020) under the Creative Commons license (CC BY). II: Reprinted from Bian et al. (2014) with permission from Elsevier, Copyright 2014. III: Reprinted from Veldhuizen et al. (2020) with permission from Elsevier, copyright 2020.

In recent years, different surface morphologies have been investigated with an aim to enhance CM maturation. For example, Kumar et al. (Kumar et al., 2020) suggested electrospun, aligned PCL fibers, covered with gelatin, with diameters of 578 ± 184 nm. It was observed that iPSCs–derived cardiomyocytes had significantly enhanced maturation markers, such as expression of cardiac troponin-T, α-sarcomeric actinin, connexin-43 (Cx-43), and synchronous calcium transients (Figure 7I). Of course, as mentioned, PCL is not environmentally-friendly, however, and selection of a green polymer would have decreased the environmental impact of such work. In a different approach (Figure 7II) (Bian et al., 2014), biomimetic molds of a human ventricle were obtained by reconstructing and replicating its ECM morphology, as visualized through magnetic resonance imaging (MRI). These molds were fabricated from polydimethylsiloxane (PDMS), via soft lithography, and the scaffolds for cell cultures were obtained by drop casting 10% Matrigel into the molds. After a 3-week culture, the as-obtained engineered tissue revealed a phenotype that was close to a mature tissue. Most notably, T-tubules with Z-disks were observed. The CMs were dense, aligned and electromechanically-coupled, and an action potential had a conduction velocity of near-adult cells. PDMS is also not environmentally friendly leaving a large carbon footprint, but it is anticipated that the same mold could be used to create green biopolymer tissue engineering materials.

In a more recent study (Veldhuizen et al., 2020), an effort to fabricate a heart-on-a chip based on microfluidics was undertaken. This more advanced study combined multiple cues: co-cultures, collagen-based scaffold, and microtopography in the form of microposts (Figure 7III). By using such a combinatorial approach, unparalleled results were obtained during a relatively short-timed culture. After 2 weeks, CMs revealed an upregulation of maturation markers (such as HCN1, KCNQ1, CAV1.2, CAV3.1, PLN, and RYR2 genes, and expression of Cx43 α-sarcomeric actinin). The cells had well-defined sarcomeric striations, were aligned and well organized, resulting in synchronized contractions. Even though the cells can’t yet be defined as fully adult, the obtained results are certainly very promising and could be further extrapolated.

## 8 Electrical conductivity

**FIGURE 8:**
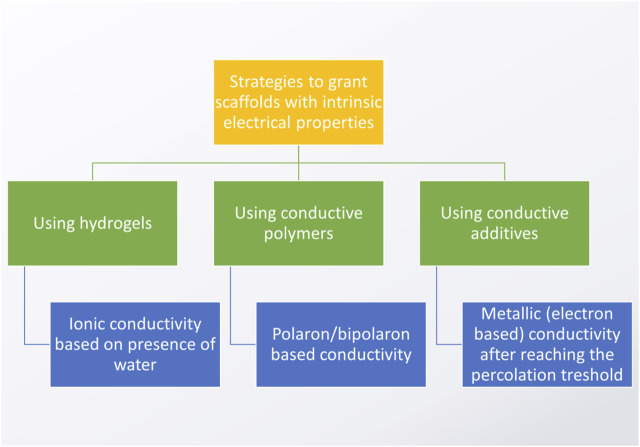
Major strategies to obtain electrically conductive scaffolds (based on the studies by: Pissis and Kyritsis, 1997; Hsu et al., 2004; Balint et al., 2014; Kaklamani et al., 2018).

There are three major strategies to obtain electrically conductive (EC) biomaterials for CVD applications (Figure 8). The simplest, but the least reliable one is based on the use of hydrogels. In these materials, ionic type conductivity is obtained by the presence of free ions dissolved in water, which is a dispersion medium. The conductivity of hydrogels is often found to be insufficient for such advanced applications as sensors or electrically-stimulated materials. Furthermore, in hydrogels, the electrical conductivity might deteriorate over time due to crystallization of salts within their structure, defragmentation of material and negative impact of elevated temperatures. For these reasons, coatings or blending with other polymers and/or electrically conductive additives/nanoadditives are usually the methods of choice when one is to obtain CVD biomaterials. It is worth noting that some scientists suggest combining two or more methods: for example, it is not unusual to see articles regarding the fabrication of hydrogel/nanoadditive (Martins et al., 2014) composites or blending with conductive polymers (Guiseppi-Elie, 2010; Walker et al., 2019), which can be further modified by combining additives with electron-based conductivity (Qazi et al., 2014; Bao et al., 2017; Wu et al., 2017). Considering the goal of facilitating cell to cell cross-talk, the conductivity of the as-obtained CVD composite material should be similar to the native myocardium, i.e. between 0.06 and 0.4 S/m (Miklavčič et al., 2006). However, when one wants to achieve further functionalities, electrical stimulation and biosensing, the higher the conductivity, the better, as it increases the efficiency of the sensing/stimulation, thus improving the material’s sensitivity and specificity. However, remember, fibers in the myocardium are anisotropic and so are its electrical properties.

The subject of fabricating electrically conductive scaffolds for cardiac tissue engineering, by using electrically conductive nanoadditives and conductive polymers, has been a focal point of a very recent and extensive review by Ashtari et al. (Ashtari et al., 2019), and the readers interested in more detailed information on the subject are kindly referred to it. In turn, the justification of using electrically conductive scaffolds for the regeneration of heart tissue, along with some important examples, are extensively elaborated on in a recent study done by Monteiro et al. (Monteiro et al., 2017). Here, we would like to provide a short guidance on what was accomplished with respect to fibrous materials recently, and what is the rationale for future perspectives of using electrically conductive environmentally-friendly cardiac scaffolds.

### 8.1 Electroconductive polymers (ECP)

The so-called conductive polymers are often polymeric materials “doped” with a dopant molecule that stabilizes their charge and is not friendly to the environment. When the conductive polymer is synthesized in its oxidized form, the dopant is positively charged, while in the reduced form, its charge is negative. If a polymer has a conjugated network, with overlapping p-orbitals, the charge can travel freely along the polymer chain and this is how electrical conductivity is achieved in this class of materials. For an elegant explanation of this phenomena, the readers are kindly referred to an excellent literature article by Balind, Cassidy and Cartmell (Balint et al., 2014).

Despite large discrepancies regarding the biocompatibility of conductive polymers and concerns regarding the environment and toxic fate of possible degradation products, there are many literature examples of their suggested biomedical applications, such as in biosensors, electrodes, drug delivery systems, bioactuators, and tissue engineering scaffolds. There are some interesting reviews on the matter and the readers are kindly referred to them for more extensive evaluation (Hardy et al., 2013; Balint et al., 2014; Yi et al., 2016; Gajendiran et al., 2017; Ning et al., 2018; Ashtari et al., 2019; Hosoyama et al., 2019). For electroconductive polymers, inherent biostability is one of the biggest challenges in their application as tissue engineering scaffolds. This quality can be modified to obtain a biodegradable polymer. The three possible routes are: 1) mixing with a degradable polymer, 2) modifying the polymer backbone and 3) synthesizing small chains that can be removed from the human body by renal clearance. Importantly, however, just because a polymer is biodegradable does not mean it is safe for use in the body or the environment. All of the above mentioned routes raise some questions regarding the fate and biocompatibility of the decomposition products which need further *in vitro* and *in vivo* studies to guarantee safe usage (Balint et al., 2014). Still, it seems that mixing conductive with biodegradable polymers may be a possible route towards the safe biomedical application of EPC-based fibrous scaffolds as it combines spinnability with degradability. This is the probable explanation why most of the recently published articles concern fabrication of such composite blends.

Currently, there are over 25 well-analyzed, electrically conductive polymers, but in cardiac tissue engineering, the ones that are the most commonly applied are polyaniline (PANi), polypyrrole (PPy) and poly (3,4-ethyl-enedioxythiophene) (PEDOT), due to their relatively low price and acceptable biocompatibility. None are considered safe to the environment and all leave a large carbon footprint. Even though PANi and PPy are electrospinnable, the conditions of electrospinning are very demanding and unsuitable for biomedical applications (for example, PANi needs to be dissolved in hot sulfuric acid). Additionally, these polymers also have unsuitable mechanical properties–they are stiff and brittle. Thus, to overcome these issues, they are most often used as coatings or blended with carrier materials and co-electrospun with other biocompatible and often biodegradable polymers, natural or synthetic: PLA, PLGA, PCL, gelatin, etc. (Balint et al., 2014). Positive results of ECP applications *in vitro* are generally attributed to their enhancement of cell to cell crosstalk (by the transmission of electrical impulses), manifested, among others, by increased expression of Cx43 protein. The addition of ECP reduces electrospun fiber diameters, most likely due to increased conductivity of the polymer solution during spinning. Cellular adhesion and proliferation are enhanced. At the same time, the synthesis of cardiac differentiation markers is improved and cellular maturation is boosted.

Still, in order to further improve the scaffold’s performance, additional cues are highly recommended. The benefits of ECP include an ability to apply electrical stimulation, which, unfortunately, has not been sufficiently exploited (less than 50% of the articles analyzed herein (Table 5, (Jeong et al., 2008; Jun et al., 2009; Fernandes et al., 2010; Yow et al., 2011; Hsiao et al., 2013; Gelmi et al., 2016; Mohammadi Amirabad et al., 2017))). It has been found that fiber alignment in CVD tissue engineering materials is also an important cue that guides cardiomyocyte growth, differentiation and maturation but only some of studies seem to be taking advantage of that fact (Ku et al., 2012; Chen et al., 2013; Hsiao et al., 2013; Mohammadi Amirabad et al., 2017), with only two exploiting both ES and fiber alignment (Hsiao et al., 2013; Mohammadi Amirabad et al., 2017). Based on the available literature, it is hard to point out which ECP would perform better as a cardiac tissue engineering scaffold–regardless of type, the presence of ECP seems beneficial but other physicochemical properties vary greatly between the studies and, thus, it is extremely difficult to ascertain which properties are enhancing cell function. Certainly, further studies are needed to be able to compare the impact of the conductive PANI, PPy and PEDOT polymers keeping all other properties the same (such as fiber morphology, diameter, alignment, mechanical properties, etc.). Furthermore, additional disadvantages regarding the usage of ECPs should be investigated, i.e. a conductivity decrease with time due to deprotonation (usually after 100 h (Hsiao et al., 2013), sometimes up to 2 weeks (Chen et al., 2013)) during which a dopant (usually acid) leaks from the sample, often causing a cytotoxic reaction. Also, environmentally-friendly materials and approaches must be investigated, even something as simple as using safer solvents–the majority of solvents used for conductive polymer synthesis are toxic. While investigating the beneficial properties of ECPs, one must also keep in mind that these polymers are not “green”—their processing requires a large carbon footprint and usage of toxic solvents. It is clear that such materials are not sustainable.

**TABLE 5:** An overview of electrically conductive polymers used to obtain fibrous scaffolds for applications in cardiac tissue engineering.

Conductive polymer	Composite used	Dopant	Rationale	Cells/*in vivo* models	Electrical stimulation	Results	Ref.
PANI	PANI/PLCL blend (up to 30% PANI)	Camphorsulfonic acid (CSPA)	• To obtain electrically conductive, elastic scaffold for the regeneration of mechano- and electro-responsive tissues •Nanofibers—biomimetic to ECM •To test the impact of PANI presence on the adhesion and viability of cells •To test the impact of ES on cellular metabolic activity	Primary fibroblasts, NIH-3T3, C2C12	Yes, for 2 days, DC from 0 to 200 mA	• Fiber diameters around 400 nm •Electrical conductivity: 0.15–1.38 S/m •Presence of PANI reduces fibers’ mechanical properties •Cells maintain normal morphology and spread evenly on the surface, their viability increases with increasing amount of PANI •200 mA caused cell apoptosis, 20 mA iproved cells’ metabolic activity (more significantly on more conductive materials) •Presence of PANI promoted myogenic differentiation in C2C12	Jeong et al. (2008); Jun et al. (2009)
	PANI nanotubes + hyperbranched poly-L-lysines (HPLys) (1.5% PANI)	Sulfuric acid	• PANI to modulate cell functions via electrical stimulation •HPLys as cardiovascular adhesives, promoting cellular infiltration	CHO cells and CM from 2 to 4 day old Sprague-Dawley rats	Yes, 8 h after seeding, for 1–96 h, AC, 10–40 V, 0.5 Hz, 5 ms pulses	• At 1.5% of PANI, fibers’ diameters around 80 nm •PANI cross-linked the hydrogel, reducing its swelling ability •PANI modified materials are biocompatible, promote CMs adhesion, proliferation and differentiation •Fibrous morphology is more beneficial than films	Fernandes et al. (2010)
	PANI/PCL blend (up to 1.5% PANI)	CSPA	• Conductive scaffold composed of aligned fibers to better mimic the ECM of skeletal muscle tissue (evaluate separated and summarized effect of these two properties)	C2C12	No	• PANI fibers’ diameter: from 280 to 500 nm for random and 230–400 nm for aligned •PANI slightly increases the Young’s modulus in aligned fibers •Increasing conductivity with increasing PANI concentration •No impact on cells’ viability, cells had elongated shapes on aligned fibers •Electrical conductivity enhanced the myogenic differentiation of cells; when materials were conductive, this effect was even furthermore increased •Electrical conductivity and alignment have synergic positive effect on C2C12 differentiation and maturation	Ku et al. (2012)
	PANI/PLGA (4.2%–8% PANI)	HCl	• Aligned fibers—biomimetic to myocardial ECM •Electrically active, aligned nanofibrous scaffolds to integrate implanted cells with the host myocardium in a synchronized manner	CM from 1–2 day old Lewis rats	Yes, time-point after seeding not specified, AC, 5 V/cm, 1.25 Hz, impulse length and shape not specified	• Beads formation and low alignment at 8% PANI •At 6.25% of PANI the fibers are uniform, approx. 100 nm thick, well-aligned and highly conductive—0.3 S/m •The materials promote cell adhesion and do not reduce their viability •Cells align with the fibers and form isolated cell clusters (beating is synchronized only within one cluster by Cx43 protein) •Electrical stimulation caused beating synchronization of cell clusters cultured on electrically conductive scaffolds	Hsiao et al. (2013)
	PANI/PCL (1%–3%)	CSPA	• Aligned and electrically conductive scaffold to provide cues for cellular attachment, elongation and differentiation	C2C12	No	• Addition of PANI improved the spinnability of PCL fibers with diameters of approx. 350 nm were obtained •The materials are highly conductive: from 1.6 to 6.4 S/m and this quality is stable for at least 2 weeks •Magnets cause efficient fibers’ alignment •Presence of PANI strengthened the nanofibers and reduced their elasticity •Cells aligned with the fibers, viability was not reduced •Cells proliferated more effectively on PANI modified scaffolds (proportional to PANI content) •Aligned and conductive nanofibrous scaffold induced muscle cell alignment and promoted myotube formation compared with the randomly oriented nanofibers •Alignment and electrical conductivity have synergic effect on the myotubes maturation and differentiation	Chen et al. (2013)
PANI	PANI/PLA (1.5% and 3%)	CSPA	• PANI/PLA fibers—biocompatible, biomimetic material to prepare a CM-based bioactuator and tissue engineering scaffold for treating myocardial infraction	H9c2 cells and CMs from 2-day old Sprague-Dawley rats	No	• Fibers’ diameters around 500 nm •PANI/PLA are biocompatible and do not influence the H9c2 cells and CMs cells’ proliferation rate •Presence of PANI resulted in enhanced length and fusion of myotubes, with >5 nuclei and fusion index at 75% as compared to 34% and 50% on pure PLA, respectively •CMs grown on PLA/PANI are more elongated and interconnected •On PLA/PANI, the CMs formed well-developed networks of sarcomeres and gap junctions, with suitability to induce synchronized beating—manifested by more synchronous, spontaneous beating, with higher beating rate •PLA/PANI/CMs create good bioactuators, with high beating frequency and maximum displacement	Wang et al. (2017)
	PANI/PES (polyetersulfone, non-biodegradable), Final PANI concentration is not specified	CSPA	• Usage of iPSC—no immunological hazard, no ethical concerns •CSPA as a dopant—high alignment, better electrical conductivity due to optimal chain packing •PANI—ROS scavenger, good electrical conductivity •PES—biocompatible, is able to induce mesodermal differentiation •PANI/PES—good mechanical stability + EC + biocompatibility •Oxygen plasma treatment to improve hydrophilicity and enhance cellular adhesion •To study the role of fiber alignment and ES on cardiac differentiation	iPSC (cardiovascular disease-specific)	Yes, AC, after seeding, 1 h a day for 15 days, 50 mV/cm, 1 Hz, square shaped, impulse length—2 ms	• Fiber diameters: approx. 270–295 nm •Highly aligned fibers are obtained at 3000 RPM •Plasma treatment reduces the materials’ mechanical properties, but these are still sufficient for the cardiac application •Conductivity up to 0.057 S/m (slightly lower than the native myocardium) •At 5th day of culture, fibrous morphology is favored for cells’ proliferation over the smooth surfaces •Electrical stimulation combined with alignment of fibers increases the number of oriented cytoskeletal fibers •In the presence of electrical stimuli and differentiating medium, aligned fibers caused improved cardiomyocytes differentiation (85%), without differentiation medium this percentage is still high (65%) •Application of electrical signals to the aligned electroactive nanofibrous scaffold produced the highest differentiation into cTnT + cells and induced the expression of cardiac-related transcription factors	Mohammadi Amirabad et al. (2017)
	PANI/PLA, PANI/PLA/PEG (2.5%–10% of PANI)	CSPA	• Conductive scaffold to mimic native ECM and transmit electrical impulses—electrical integration with damaged tissue •PLA—easy processing ability, PEG –high biocompatibility, plasticizer, PANI—high electrical conductivity •Simple and core-shell fibers	NRK, MCF-7, and MG-63 for cytotoxicity, cardiac fibroblasts and cardiomyocytes from 1 to 3 day-old mice for biocompatibility	No	• Fiber diameters 2–5 µm •Relatively low conductivity: 0.0001 S/m •For simple fibers: above 2.5% of PANI, the materials are cytotoxic and presence of PEG enhances this effect by facilitating PANI release into the media; core-shell fibers are less cytotoxic •Cardiomyocytes adhere and proliferate on fibers, conforming to their morphology, beating starts at 7th day of culture (not observed on pure PLA)	Bertuoli et al. (2019)
PPy	PPy/PCL/gelatin (15% and 30%)	Not specified	• Electrospun fibers and conductive fibers—biomimetic to ECM •PPy—higher conductivity •PCL—to improve mechanical properties and grant biodegradability •Gelatin to improve biocompatibility	Cardiomyocytes isolated from New Zealand white rabbits	No	• Fiber diameters 190–240 nm •PPy increases the material’s hydrophobicity and Young’s modulus •30% PPy fibers conductivity of 0.037 S/m (close to native myocardium), but too fast degradation rate •PPy improves cells’ proliferation rate and enhanced Cx43 synthesis—improved electrical and chemical coupling between the cells, enhanced differentiation	Kai et al. (2011)
	PPy/polyelectrolyte complexation (PEC) fibers from methylated collagen	FeCl_3_	• PEC: bioactive and biocompatible but poor mechanical properties •PPy to improve the mechanical properties and introduce electrical properties •Fibrous scaffold for the regeneration of electrically active tissues	hMSCs	Yes, AC, square biphasic waves of 1.2 V, 5 ms pulse duration, 200 Hz frequency	• PPy formed beads inside the PEC fibers and the fibers thinner than 3 µm •PPy improved mechanical properties of the fibers •At day 5th ES improved the proliferation rate, but retarded it at day 10 •Under the experimental conditions, enhanced differentiation into neural lineage was achieved	Yow et al. (2011)
PPy	PLGA coated with PPy layer (320–490 nm thick)	Dodecylbenzenesulfonate (DBS)	• Fibrous scaffold to increase stem cell survival rate, enhance retention and improve cardiac functionality (as compared to injected stem cells) •When electrically stimulated, PPy changes volume, thus performing mechanical and electrical stimulation to enhance scaffold’s performance •Usage of DBS as a dopant to avoid leakage •iPS cells—developed from patient-derived cells to reduce the risk of immune rejection	iPSC	Yes, AC, biphasic potential (0.2 V to −1 V) at three frequencies (0.05, 0.1, and 0.2 Hz)	• Diameter of PLGA fibers: 2.27 µm, after coating: 2.91 (10 min); 3.24 (30 min) •PLGA start to degrade after 7 days but the layer is intact with conductivity preserved •Only the thicker layer was able to mechanically actuate and the highest cyclical actuation was seen at 0.05 Hz stimulation •All of the materials are non-toxic and the thicker layer is suggested to enhance the iPS differentiation along cardiac lineage	Gelmi et al. (2016)
	PPy *in situ* polymerized on mussel-derived chitosan scaffold	FeCl_3_	• PPy to sustain the electrophysiological maturation and functionality of CMs, guiding regular beating of the heart with low cytotoxicity •Chitosan—minimal immune reaction •Mussel shell derived chitosan—3D architecture	**Cells:** Neonatal rat ventricular myocytes isolated from 1 to 3 day old Sprague–Dawley rat *In vivo* model: Male Sprague–Dawley rats with permanent left anterior descending (LAD) ligation, pre implantation, scaffolds were seeded with CMs	No	• PPy uniformly coated the porous structure, maintaining open porosity, increasing roughness and increasing material’s stiffness •Conductivity as high as 0.07 S/m (within the range of native myocardium) •The material had suitable degradation rate (24% mass decrease after 6 weeks), with stronger elongation and more elastic and ductile properties after incubation, when compared to pure chitosan •PPy modified material is biocompatible, promotes CMs adhesion, elongation, maturation and cell-cell coupling •At day 3 of culture, PPy scaffold displayed the whole contraction with the largest amplitude and linear displacements •*in vivo* mean fractional shortening and ejection fraction were significantly increased, enhanced thickness and contractile activity—materials maintained heart function and accelerated cardiac repair with enhanced cardiomyogenesis and angiogenesis	Song et al. (2019)
PEDOT	Chitosan/PVA/PEDOT:PSS (0.3, 0.6 and 1 wt%)	Not specified	• Conductive fibers to mimic the natural cardiac ECM •Chitosan: high biocompatibility, modified with PVA to facilitate electrospinning •PEDOT:PSS—higher chemical and thermal stability than PPy and PANI, good biocompatibility	Rat bone marrow mesenchymal stem cells	No	• Fibers with diameters from 84–117 nm (inversely proportional to PEDOT:PSS content), with higher mechanical properties than pure chitosan/PVA •Electrical conductivity as high as 0.008 S/m (one order of magnitude lower than native myocardium) •The materials were biocompatible, supported cell adhesion and proliferation	Abedi et al. (2019)

Due to some issues with long term stability and possible toxicity, it is hard to find *in vivo* studies testing the materials’ actual performance in treating damaged cardiac tissue. In fact, for this review, we were not able to find any *in vivo* studies done on conductive fibrous scaffolds as described herewithin. In 2018, Wang et al. (Wang et al., 2018) investigated the impact of injectable hydrogels based on tetraaniline, while a 2019 study by Song et al. concerned 3D porous scaffolds modified with PPy (Song et al., 2019). In both studies, the applied materials were able to maintain heart function and accelerate cardiac repair with enhanced cardiomyogenesis, angiogenesis and maturation. One can only imagine that endowing the materials with additional biofunctional cues (i.e. electrical, topographical, and/or mechanical stimulation with an aligned nanofibrous architecture) could yield even better results.

### 8.2 Nanoadditives

Blending polymers with electrically conductive additives is not a new subject. In fact, it dates back as far as the 1950s, when one of the first patents was filed (Cole, 1956). The invention assumed covering polymer particles used for molding with metallic films. In the 1990s, this idea was evolving and many researchers mixed polymer powders with conductive fillers, most notably, carbon black. Back then, it was already well understood that proper distribution is key to reaching an optimal conductivity with the lowest possible concentration of the additive (Ouyang and Chan, 1996). Most commonly, the electrical conductivity of composites related to the presence of the filler is described by the percolation theory and percolation threshold (p_t_). From the material scientist’s point of view, p_t_ is the lowest possible concentration of the filler at which it is able to form a “connected component”—a network of connected particles through which electrical charge is able to travel freely. Generally, the higher the particle surface area (and, thus, smaller size), the easier it is to reach a percolation threshold–hence, smaller amounts of nanofillers are needed to yield electrically conductive materials, when compared to micro and macro-scaled additives. Higher amounts are uneconomical and could deteriorate a material’s mechanical properties. Additionally, some of the additives may evoke a toxic reaction at higher concentrations. For better understanding of the percolation threshold phenomenon, the reader is kindly referred to a recent study by Saberi (Saberi, 2015). To sum up, using nanoparticles is beneficial as lower amounts need to be added to the matrix to reach the certain conductivity, thus, representing a green approach. Additionally, when considering fabricating nano-sized morphologies, one is forced to also scale down the components’ size. For these reasons, the subject of fabricating electro conductive scaffolds is currently dominated with nanofillers, most notably carbon and gold. The main focus is to find a highly conductive filler that is biocompatible, easily dispersible in a polymer matrix and has a high surface area, reducing the percolation threshold (Mao et al., 2012). Morphology is also important–when the particles have elongated shapes (fibers, tubes), their alignment within the matrix can be forced during processing and may lead to a further increase in material properties (Ahadian et al., 2016). The two major approaches to introduce electrical conductivity to the scaffold by means of using conductive nanoparticles are: creation of a conductive layer (Zhao et al., 2018) or use of matrix modificators (Kitsara et al., 2017). While the former will generally grant higher surface conductivity, a risk of flaking resulting in an uncontrolled release of particles and electrical properties deterioration are some of the reasons why matrix modification is more common. Additionally, using a properly selected matrix modification may enhance not only the material’s electrical behavior but also its mechanical properties. For these reasons, the following paragraphs will focus mostly on matrix modification of scaffolds to enhance conductivity.

#### 8.2.1 Questions of toxicity

**FIGURE 9:**
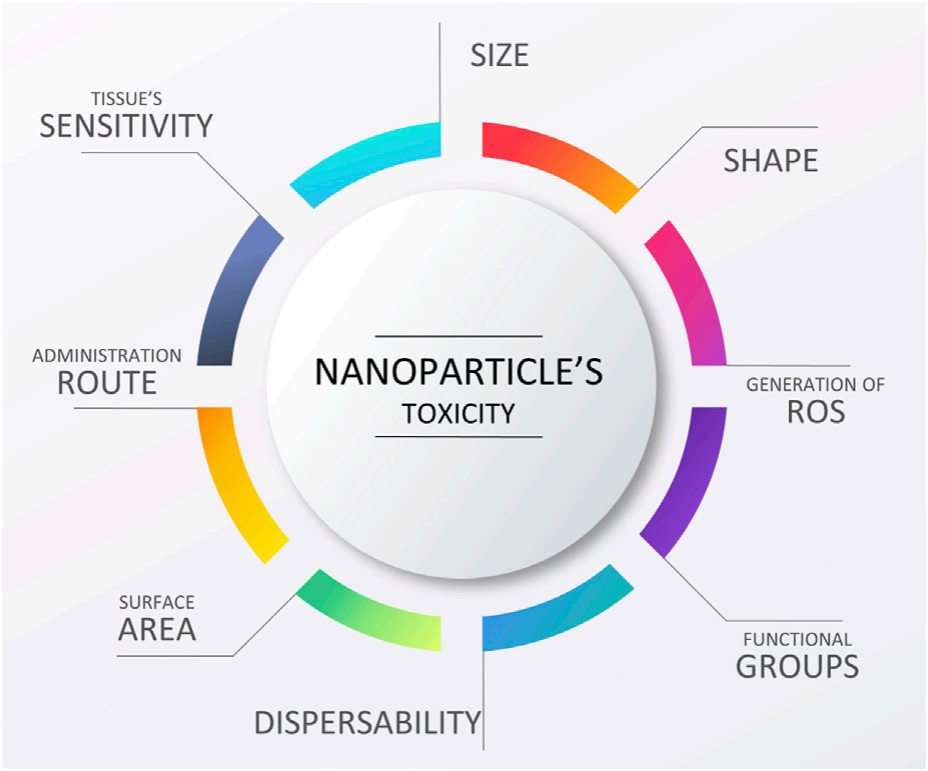
Factors affecting nanoparticle toxicity/biocompatibility, graphical scheme designed by TinyPPT.com.

When reducing the size of a conductive additive, a question of its potential toxicity becomes more and more burning. This is due to the fact that the nanoparticles can interact directly and indirectly with cells in numerous ways, damaging their structure, changing their metabolism and reducing their viability (Benko et al., 2021; Benko et al., 2023)—all of which can have negative consequences, starting at irritation and inflammation, and ending at necrosis and formation of malignant cancers (Shvedova et al., 2012; Vial et al., 2017). This is a very complicated matter as the occurrence of unwanted reactions depends not only on the nature of the nanoparticle, but also its chemistry and how it is introduced into the body and/or environment. While in classical biomaterial science, some substances are inherently toxic or non-toxic, in the case of the nanoparticles, there is a plethora of physicochemical properties that affect cellular reaction: such as size, shape, state of surface, dispersability, specific surface area, tissue specific behavior, administration route and overall an ability to create reactive oxygen species (ROS) (Figure 9). Specifically, surface related characteristics become more important as the surface-area-to-volume ratio becomes unproportionally higher than in micro and macro sized biomaterials. The complexity of the matter, together with an insufficient physicochemical evaluation of the nanoparticles used by different scientists, has led to large discrepancies concerning the safe application of nanomaterials, among which carbon nanotubes are likely the most controversial. However, careful evaluation of available data indicates that safe usage of nanoparticles may be achieved by using particles clean of metallic catalyst, with low aspect ratio, two dimensions exceeding 15 nm with small and polar functional groups covalently attached to the surface enabling good dispersion in body fluids, followed by facilitated clearance or even biodegradation. Additionally, target cells as well as form of application and administration route should always be carefully selected to be able to evoke specific reactions (Kostarelos et al., 2007; Kostarelos, 2008; Vittorio et al., 2009; Kotchey et al., 2013; Bhattacharya et al., 2016; Chatterjee et al., 2016; Zhang et al., 2016; Vial et al., 2017). Please do note that while some studies have been completed on *in vivo* toxicity to nanoparticles, much less research has been conducted on environmental toxicity of nanoparticles. This needs to be fully understood before nanoparticles can be viewed as environmentally-friendly as most likely such properties will depend on the chemistry of the nanoparticles, how they were synthesized, and how they were introduced into the environment.

#### 8.2.2 Gold nanoparticles (GNPs or AuNPs)

Gold as a biomaterial has one of the oldest histories of use, with well-established biocompatibility manifested by at least a few thousand years of biomedical applications (Ratner et al., 2013b; Vial et al., 2017). From this point of view, gold nanoparticles are relatively young as their medical applications date back only to 1971, when they were applied in immunochemistry by Faulk and Taylor (Dykman and Khlebtsov, 2011). Progressing from the 70s, a plethora of possible medical applications have emerged: from cell therapies and drug delivery systems, through labeling tools, ending at scaffold modification. In tissue engineering applications, GNPs may be used to improve mechanical properties, enhance cellular adhesion or grant the material with electrical conductivity. GNPs can be synthesized in various shapes and sizes, tailored to meet specific applications (Dykman and Khlebtsov, 2011; Vial et al., 2017).

In cardiac tissue engineering, the idea to use gold nanoparticles started around 2011 with numerous scientists proving its excellent cytocompatibility and ability to promote desired cellular behaviors, such as improved cell adhesion, elongated morphology, faster proliferation and improved maturation of both adult and stem cells-derived cardiomyocytes. Most scientists suggest that the superior biological response of gold nanoparticles is due to its improved cell to cell cross-talk (due to electrical conductivity) and altered nanotopography (Vial et al., 2017; Ashtari et al., 2019).

In 2016, several scientific groups independently studied the beneficial impact of GNP modified polymers (both natural and synthetic) on cardiac lineage cells (Table 6). When combined with electrical stimulation, materials improved cellular adhesion, proliferation and differentiation. Cells were found to possess an elongated morphology. Forming well packed tissue-like constructs, cardiac cells revealed more synchronized beating with a reduced excitation threshold (Baei et al., 2016; Ganji et al., 2016; Navaei et al., 2016; Thrivikraman et al., 2016). Remarkably, Thrivikraman et al. induced stem cell differentiation along the cardiac lineage without using any exogenous biochemical factors but by combining GNPs inside cells and physiologically electrically stimulating them (Thrivikraman et al., 2016). However, it should be noted that the studies were conducted on human mesenchymal stem cells for which differentiation into CMs should be treated with great caution–their differentiation potential will depend on the cell source and it is generally acknowledged that these cells have the ability to form cardiomyocyte-like cells and not cardiomyocytes (Guo et al., 2020). This is due to elevation of some (but not all) CM-specific markers. Studies on hydrogel scaffolds revealed a beneficial impact of gold on the material’s mechanical properties (Li et al., 2016; Zhu et al., 2017). Interestingly, Li et al. (Li et al., 2016) found that GNP-modified scaffolds improved biofunctional properties, as compared to pure collagen, upon seeding with neonatal rat ventricular myocytes. The team hypothesized that improved maturation of cells and faster assembly of intercalated discs can be attributed to changes in nanolocal stiffness, emphasizing an importance of mechanical nanocues in cardiac tissue regeneration. However, in order to separate GNP presence (altering, among others, roughness and electrical conductivity) from their impact on mechanical properties, more studies should be performed.

**TABLE 6:** Selection of GNPs used in cardiac tissue engineering.

Year	Additive (concentration)	Polymer	Morphology	Rationale	Cells	Electrical stimulation	Results	Ref.
2016	Gold nanorods, with increasing concentration (∼1–3% w/w)	GelMA	Porous scaffold	• Polymer for ECM-like environment and GNPs to improve adhesion and grant the material with electrical conductivity	Neonatal rat ventricular cardiomyocytes	• Yes, according to Tandon’s protocol Tandon et al. (2009), •AC, square wave, pulse duration 2 ms, 1, 2, 3 Hz	• GNRs did not change GelMA’s micro-structure • GNRs at the highest concentration improved material’s conductivity and stiffness, and decreased swelling On GNR modified • Higher number, more packed and homogeneous distribution of adhered cardiomyocytes • Better cell retention, leading to higher viability and formation of a uniform and interconnected tissue layer • Organized myofilament assembly and improved cell-cell coupling with mature contractile machinery • Higher beating frequency, with better synchronization, stability and robustness • Reduced excitation threshold	Navaei et al. (2016)
2016	GNPs (1%–1.5% w/w)	Chitosan	Injectable hydrogel	• Chitosan—a biocompatible, thermosensitive hydrogel to deliver stem cells • GNPs to grant it with electrical conductivity • Together—electro-conductive injectable hydrogel mimicking the electromechanical properties of the native myocardium	Human bone marrow derived MSCs	• No	• Electrical conductivity 0.16 S/m • GNPs induced and enhanced differentiation of MSCs into CM-like cells without any adverse effect on the hydrogel’s physiomechanical properties	Baei et al. (2016)
2016	GNPs (concentration not specified—layers)	PANi layer	Internalized GNPs, GNPs layers of PANI	• GNPs—good cellular uptake and high conductivity useful for electrical stimulation	hMSCs	• Yes, for 14 days	• GNPs are readily internalized by cells and found biocompatible • A combination of intracellular GNPs and conductive substrates influenced the transformation of hMSCs to neural-like cells • 1 Hz frequency stimulated cellular elongation, accompanied by nuclear shape change and desmin expression in cells with internalized GNPs • Physiological conditioning with pulsed electric field in conjunction with intracellular GNP induces cardiomyogenesis on any surface	Thrivikraman et al. (2016)
						• Neurons: DC, 100 mV/cm, 15 min/day		
						• Cardiac cells: AC square wave, 10, 1 and 0.1 Hz, with a duty cycle of 10% and field strength of 100 mV/cm for 15 min/day		
2016	Gold nanotubes/nanowires (50–100 ppm)	Polyurethane	Sponges modified in mass with gold particles (salt-leaching technique)	• Biodegradable polyurethanes—good biocompatibility and biodegradability, high flexibility, excellent mechanical properties (Young’s modulus similar to heart –from 10 kPa in the beginning of the diastole to 500 kPa at the end of the diastole). Compressive modulus of native heart—425 kPa at the systole • Gold to improve the transmission and synchronization of electrical signals in the material and thus increase the natural functionality of cardiomyocytes • Scaffold is to mimic the electromechanical properties of the myocardium	H9C2	• Yes	• GNPs changed the physicochemical properties of PU and improved cell attachment • Before stimulation, no significant difference in cell morphology and proliferation • Cell prefer adhering to 50 ppm NPs, but electrical stimulation causes increased adhesion to 100ppm too • Electrical stimulation induced cell alignment on conductive materials in the direction of the applied field • PU-GNT/NW scaffolds can accelerate cardiomyocyte response to the stresses induced by electrical stimulation, decreasing the progress of cardiac hypertrophy • 50 ppm of GNT/NW is an optimal concentration for stimulating the expression levels of important cardiac differentiation markers and of myogenesis	Ganji et al. (2016)
						• Square pulse of 1 V/mm amplitude, pulse duration of 2 ms, at a frequency of 1 Hz for 15 min		
2016	Gold nanoparticles (0.05–0.2 mg/ml, w/w concentration not specified)	Collagen	Layers on glass slides	• Collagen—biocompatible, can be harvested from the patient • GNPs to improve mechanical properties • Local nanoscale stiffness and nanotopography to increase the interaction between cardiac myocytes and scaffold	Neonatal rat ventricular myocytes from Sprague–Dawley rats	• No	• GNPs scaffolds significantly improved the development and maturation of cardiomyocytes with no negative effect on viability and metabolic activity • Cells grown on GNPs-scaffold were more elongated and aligned • GNPs promoted assembly of intercalated discs • GNPs changed nanolocal stiffness, improving cellular adhesion	Li et al. (2016)
2017	Cetyltrimethylammonium bromide (CTAB)-coated gold nanorods (c-GNRs), coated with GelMA (0.2%–1% w/w)	GelMA/alginate	Bioink for 3D printing with cells	• GelMA/alginate—good bioprinting hydrogel • Gold to grant material with electrical conductivity and to attenuate the overproliferation of CFs • 3D printing—co-print materials with cells for better cellular infiltration and desired morphology	Cardiomyocytes and cardiac fibroblasts (CFs)	• No	• The biocompatible bioink can be used to print 3D scaffolds of desired morphology • Material is found to improve expression of gap junction protein and enhance synchronized beating of cells	Zhu et al. (2017)
2017	Gold nanoparticles (round, 15 nm), amine functionalized (not specified, layer)	Porcine cholecystic extracellular matrix (C-ECM)	Gold nanoparticles deposited on the surface of naturally derived fibrous ECM	• Gold nanoparticles to improve scaffold homogeneity	H9C2	• No	• Deposition causes formation of thicker, more uniform fibers • Material is non-toxic, favorable for cell adhesion and proliferation	Nair et al. (2017)
2018	Gold nanorods (GNRs) (not specified)	Amyloid fibrils	Gold nanorods deposited on amyloid fibrils	• Amyloid fibrils = mechanically and chemically strong, biomimetic scaffold	None	• No	• Successful fabrication of a highly conductive scaffold	Taheri et al. (2018)
				• GNRs to grant conductivity				

Even though gold nanoparticles are quite often incorporated into scaffolds, benefits of their exclusive presence have not been carefully reported or investigated, i.e., electrical conductivity of gold-modified materials has not been well studied as electrical stimulation has not been applied, which is somehow surprising given the excellent results reported by Thrivikraman et al. and their successive review article on that matter (Thrivikraman et al., 2016; Thrivikraman et al., 2018). What is more, the morphology of the scaffolds is often not biomimetic to the native myocardial ECM–it is not fibrous. We strongly believe that progress in the treatment of myocardial infraction cannot be made without combining multiple biofunctional cues to induce a desired level of proliferation, differentiation and maturation into fully functional, adult tissues. What is more, the GNPs used in most of the studies are often non-dispersible in their un-modified state and their fate during uncontrolled or controlled release from the scaffold during its degradation tends to be neglected. It is our opinion that before the GNPs are to be used in clinics, their long term *in vivo* toxicity, with respect to their aspect ratio, shape and tendency to agglomerate, should be evaluated, similar to the multiple studies that were done on CNTs within this field (Kostarelos et al., 2007; Kostarelos, 2008). Moreover, protocols to grant the materials with long-term stability in polar fluids, as to facilitate the biological clearance, should be established. Further, although there are green chemistry methods to produce gold nanoparticles, most (if not all) of the gold nanopartilces used for CVD applications found in the literature are made through the use of toxic chemicals.

#### 8.2.3 MXene

MXenes are a relatively new class of 2D materials, formed with an atomically thin layer of transitional metal carbides and nitrides. The first mention of MXenes date back to 2012, and recently, they have been attracting increasing attention in biomedical applications (Rafieerad et al., 2021; Bashandeh et al., 2022). This is because they are relatively easy to mix with various matrix materials, they are biocompatible, antibacterial, and can provide composites with electrical conductivity. In a recent study by Asaro et al. (Asaro et al., 2023), composites based on collagen modified with gelatin-modified MXene were fabricated. The product improved electrical conductivity and was found to facilitate CM adhesion, spreading and elongation. During *in vitro* cultures which were aided with electrical stimulation (biphasic, 2 ms long pulse, ±2.5 V amplitude, and a frequency of 2 Hz, 1 h/day for 4 days), iPSC-CMs cells enhanced expression of Cx43, indicative of a more mature phenotype. While these are preliminary results and more analyses is needed to confirm the findings, this is certainly an interesting an exciting new class of materials to be used in cardiac tissue engineering. It also remains to be seen how environmentally friendly or unfriendly MXenes are.

#### 8.2.4 Carbon nanoparticles (CNPs)

Since Eiji Osawa’s study in 1971 that predicted the existence of a new carbon nanoform (fullerene) followed by its successful synthesis in 1985, nanoforms of carbon have been attracting significant attention in numerous fields of science, due to their incredible mechanical and electrical properties, high potency for chemical modification, and small size (Kratschmer et al., 1990; Smalley et al., 1998; Boyd and Slanina, 2001; Delgado et al., 2008; Aziz et al., 2011). For almost 40 years, studies have elucidated their unique physicochemical properties and have inspired multiple applications, including medical, upon which many extensive reviews have been written (Saito et al., 2011; Nunes et al., 2012; Fraczek-Szczypta, 2014; Saito et al., 2014; Gonçalves et al., 2016; Serpell et al., 2016). These point out that careful tailoring of carbon size, surface chemistry or structural properties is a route towards safe and effective biomedical applications, including those for CVD. By this means, tissue engineering materials with a unique impact on cells can be obtained: improving cellular adhesion, enabling cellular interactions, guiding and enhancing stem cell differentiation, and providing a possibility for electrical stimulation (Stout and Webster, 2012). In cardiac tissue engineering, the benefits of carbon nanoforms use has been analyzed in great details in the review by Dozois et al. (Dozois et al., 2017). In the following sections, we’d like to provide some more examples of such excellent results, dividing the carbon nanoforms by their structure. Importantly, while initial carbon structures were formulated through environmentally unfriendly methods (such as the use of methane and/or chemical vapor deposition), new efforts have revealed that carbon materials can be made from and found in numerous natural sources, such as microwaving honey to form carbon nanoparticles.

##### 8.2.4.1 Carbon nanofibers

Carbon nanofibers (CNFs) are miniaturized forms of traditional carbon fibers (CFs) which have been analyzed for potential biomedical applications since the 1970s. Due to their excellent mechanical properties, low density and high inertness, traditional applications of CFs are in orthopedics (such as for tendon and ligament replacement, bone and cartilage tissue engineering, spinal implants, etc.) and other applications, including left-ventricular assist devices, tracheal prosthesis, or hernia meshes; all have reported excellent results (Saito et al., 2011). Thanks to progress in fabrication methods, CNFs can now be easily synthesized (most notably, via electrospinning (Stodolak-Zych et al., 2016; Benko et al., 2019)). They are regarded to be more interesting for biomedical applications than their micro-sized cousins, due to higher surface area, improved electrical conductivity and higher morphological biomimetism to natural tissue fibers (Elias et al., 2002; Price et al., 2003; Tran et al., 2009; Saito et al., 2011; Zhang et al., 2014). When it comes to cardiac tissue engineering, a series of studies in the Webster group by Stout et al. and Asiri et al. have proven that human cardiomyocytes prefer CNFs-modified composites over the unmodified polymers (manifested by improved cell adhesion, proliferation and metabolic activity), due to increased surface roughness and better electrical conductivity, with CNF alignment further enhancing these effects (Stout et al., 2011; Stout et al., 2012; Asiri et al., 2014). In another study done by Martins et al., the addition of CNFs into a chitosan scaffold resulted in materials with conductivity similar to the native myocardium, which was able to enhance cardiogenic properties of neonatal rat CMs (Martins et al., 2014) (Table 7). Despite these promising results, more recent reports on CNF applications in cardiac tissue engineering are hard to find. It seems that CNTs and graphene have taken over this field, most likely due to the higher availability of such materials with different physicochemical properties, including the ones that are more dispersible in polar liquids, and hence potentially safer in biomedical applications. CNTs and graphene are also more versatile and easier to work with than CNFs. None-the-less, they all show promise for CVD applications.

**TABLE 7:** Carbon nanofibers used in cardiac tissue engineering.

Year	Type of additive	Polymer used	Morphology of matrix	Application	Rationale for this composition	Cells used	Electrical stimulation	Results	Citiation
2011, 2012, 2014	CNFs	PLGA	Nanofibrous scaffolds and films on glass slides	Myocardial infraction	• To understand why cardiomyocytes prefer some nanocomposites over others • CNFs to enhance cellular adhesion and provide better mechanical and electrical properties	Human cardiomyocytes	No	• CNFs increase the material’s hydrophobicity and reduce the degradation rate • Material has maximum conductivity: 0.16 S/m, and improved mechanical properties • Increased surface area up to 50% share of CNFs resulted in higher protein adsorption • Presence of CNFs enhances cardiomyocytes adhesion, proliferation and metabolic activity • Cardiomyocytes prefer conductive materials with aligned, fibrous features	Stout et al. (2011); Stout et al. (2012); Asiri et al. (2014)
2014	CNFs	Chitosan	Porous scaffolds	Cardiac tissue engineering	• Electroconductive scaffold	Neonatal rat cardiomyocytes	No	• CNFs enhance conductivity to reach 0.03–0.6 S/m • Enhanced cardiogenic properties (elevated metabolic activity, higher gene expression) without exogenous electrical stimulation	Martins et al. (2014)

##### 8.2.4.2 Carbon nanotubes

Carbon nanotubes are multiple (multi-walled CNTs, MWCNTs) or single (SWCNTs) rolled up sheets of graphene. Depending on the rolling axis (chiral vector), the chiral, arm-chair, or zig-zag configuration of the tube can be obtained. Different structural orientation yields varying mechanical and electrical properties, with arm-chair CNTs having lower critical tensile strain and metallic type conductivity, while zig-zag and chiral–higher critical tensile strain usually have a semi-conducting character (Mori et al., 2005; Sanchez-Valencia et al., 2014). It is estimated that the synthesis of SWCNTs yields a mixture of equal amounts of three types of configurations (not to mention possible imperfections in the structure)—this can cause issues in the reproducibility of the results, as with every usage, different amounts of each type of CNTs can be taken. Meanwhile, MWCNTs which are made of multiple layers of graphene sheets, forming tubes of varying chirality, are generally more uniform in their physicochemical properties, with metallic type conductivity and higher mechanical properties than SWCNTs. Another advantage of MWCNTs is their size–having diameters usually exceeding 15 nm, these CNTs have lower tendency to become tangled and agglomerated and are generally less toxic (Mori et al., 2005; Eres et al., 2010; Chatterjee et al., 2016). These are the main reasons why MWCNTs are more often used in biomedical applications than SWCNTs, and with better results (Dozois et al., 2017). For example, in 2011, Sirivisoot et al., found that myocytes preferred MWCNTs-modified polyurethanes over SWCNTs-modified ones, as manifested by increased proliferation rate, further improved by electrical stimulation (Sirivisoot and Harrison, 2011). Compared to other conductive additives, CNTs with their tubular shape have higher biomimetism to the natural ECM, have excellent mechanical and electrical properties, and are of high commercial availability (coming in different shapes, varying in surface chemistry, etc.). What is more, recent results suggest that highly functionalized CNTs may be degraded after internalization by immune cells, promising higher safety in applications in tissue engineering scaffolds (Kotchey et al., 2013; Bussy et al., 2016; Modugno et al., 2016). Further, recent research has shown that CNTs can be made without toxic catalysts and even with plants and natural materials representing an environmentally-friendly material.

Since the 2005 study by Garibaldi et al. which reported excellent CNT biocompatiblity properties with cardiomyocytes (Garibaldi et al., 2005), applications of CNTs in cardiac tissue engineering have gained increasing attention with very promising results (see Table 8 for a short selection, while more extensive information can be found in reviews by Ashtari et al. (Ashtari et al., 2019), Gorain et al. (Gorain et al., 2018) and Dozois et al. (Dozois et al., 2017)).

**TABLE 8:** Selection of CNTs used in cardiac and muscle tissue engineering.

Year	Type of additive	Polymer used	Morphology of matrix	Application	Rationale for this composition	Cells used	Electrical stimulation	Results	Citiation
2011	SWCNTs and MWCNTs (0.2% w/v of polymer solution)	Polyurethane	• Electrospun, fibrous scaffold	Muscle tissue engineering	• Fibers—biomimetic •CNTs—conductivity to enhance regeneration	C2C12	Yes, 22 V/cm, 20 Hz, 1 ms pulse width	• Increased hydrophilicity •Enhanced ultimate tensile strength and stiffness •Conductivity of 0.15 S/m •Increased cell density •MWCNTs increased the number of myotubes to a higher extent •ES increased the number and length of myotubes, most significantly on MWCNTs	Sirivisoot and Harrison, (2011)
2014	MWCNTs 2 conc.: 2.5% and 0.25%	Gelatin	• Aligned fibers	Skeletal muscle tissue engineering	• Gelatin - biodegradable, biocompatible and cell supportive properties •MWCNTs improve the mechanical, biological, and electrical properties •Alignment to mimic the natural ECM architecture	C2C12 skeletal muscle	2 days of electrical pulse stimulation of 5 V at a frequency of 1 Hz and for 1 ms, Ion Optix, frequency <20 Hz promotes the formation of slow fiber type and between 50–150 Hz—the fast fiber type	• MWCNTs increase the fibers’ diameters •Two-fold increase in Young’s modulus •The obtained materials are insulators •Cells align to the fibers, within 1 h after seeding, by the means of contact guidance •Myotubes with lengths of 150 μm, 267, and 630 μm formed after 4 days in differentiation medium for gela, 0.25% CNTs and 2.5% CNTs, respectively •ES caused a 45%, 42% and 13% enhancement of the myotube length after 2 days of culture, respectively •Higher maturation and contractibility of myotubes with increasing the MWNT concentrations	Ostrovidov et al. (2014)
2016	MWCNTs	PEDOT	PEDOT-coated MWCNTs fibrous sheets with differentiated C212 cells seeded on them—either uniformly or in the hornworm morphology (artificial muscle)	• Fabrication of hybrid muscle actuator	• MWCNTs are electrically conductive, promote cellular adhesion and induce self-alignment of myotubes •PEDOT increases the material’s stability and ease to handle, increases the hydrophilicity	C2C12 skeletal muscle	• Yes EFS with a 10 ms step pulse duration (60–80 V) were applied at a variable frequency of stimulation (0.5–4 Hz)	• PEDOT/MWCNTs revealed high differentiation efficiency of C212 cells •Highly aligned myotubes are formed •Electrical stimulation causes contraction of single myotube and the artificial muscle •Shape-maintenance property of the PEDOT/MWCNT sheet does not hinder muscle contraction, but assists its relaxation by reducing the inner energy consumption •The material can be used as a culture platform for a variety of cell types •Electrical conductivity of the PEDOT/MWCNT platform allows bidirectional electrical communications between the attached cells (muscle and nerves) and the platform	Kim et al. (2016)
				• Patch on artificial organ					
				• Biosensor					
2016	Oxidized MWCNTs	GelMA	GelMA hydrogels with random and aligned CNTs	Creating biomimetic stem cell niches *in vitro* for controlling stem cell behavior in therapeutic applications	• CNTs to enhance scaffold’s flexibility, strength, and electrical conductivity •Hybrid GelMA-CNT hydrogels with tunable electrical and mechanical characteristics to culture and electrically regulate the cardiac differentiation of mouse embryoid bodies •CNTs alignment - better performance in the generation of functional and contractile skeletal muscle myofibers	129/SVE-derived mouse stem cells	• Yes •Electrically stimulated along CNT alignment at day 2. The ES was applied using a waveform generator: (frequency, 1 Hz; voltage, 3 V; duration, 10 ms), continuously for 2 days.	• The CNTs significantly increased the stiffness of pure GelMA hydrogels •The EBs cultured on the GelMA-aligned CNTs differentiated more towards cardiomyocytes in contrast with the EBs on the pure GelMA and GelMA containing randomly oriented CNTs. This effect was more pronounced when the ES was applied •Elevated cTnT, Nkx-2.5, and ACTC1 genes in aligned and es-stimulated cnts-modified materials •EBs seeded on the GelMA hydrogels containing the aligned CNTs had greater beating activity, further increased by ES	Ahadian et al. (2016)
2016	CNTs (most likely SWCNTs but not specified)	Reticulated vitreous carbon (RVC) foams Carbon fibers	Foams covered with CNTs and Si fibers covered with CNTs	Regeneration of skeletal muscles	• Immobilization of CNT carpets on Pristine-Foams through strong interaction with underlying silica substrate nanolayer will prevent shedding of individual CNTs and minimize their cellular uptake •To increase the surface roughness •To enhance wettability	C2C12	• No	• Nano-functionalization with CNT carpets enhanced cell-Foam interactions and secretion of ECM, while being cytocompatible •CNTs functionalization promoted differentiation of myoblasts into myocytes •Nanoroughness, rather than wettability is more important in controlling myoblast differentiation into MHC-positive cells •Aligned fibrous mats are more efficient in promoting differentiation at an earlier time point than porous foams •Presence of CNTs on fibers promoted efficient fusion of differentiated myocytes to form myotubes, forming continuous myotube bundles •CNT-Fiber significantly enhanced myocyte fusion into multinucleated and mature myotubes, highlighting the synergy between surface nano-topography and aligned fibrous architecture	Patel et al. (2016)
2017	Oxidized MWCNTs	124 polymer	Porous scaffold in the mesh form	Cardiac tissue engineering	• CNTs for conductivity and structural support •124 polymer—highly elastic, biocompatible	Cardiomyocytes (CMs) isolated from the hearts of 2-day-old Sprague−Dawley rats	• No	• Faster development on consistent and spontaneous beating on CNTs-modified •Higher concentration = more coordinated beating	Ahadian et al. (2017)
2017	CNTs (no physicochemical information)	Silk fibroin and GelMA sponges + PCL/silk fibroin + CNT yarn (wet-dry electrospinning)	1 and 2 layer scaffold—PCL/SF/CNTs weaved with suturing threads and covered with GelMA sponge	Cardiac tissue engineering	• To mimic the complex 3D anisotropic structure of heart	Cardiomyocytes (CMs) isolated from the hearts of 2-day-old Sprague−Dawley rats (on fibers) + endothelial cells (EC) (on hydrogel shell)	• No	• Fibers are biocompatible and guide CMs cellular alignment and elongation, and enhance CMs maturation and function •In multilayered scaffolds, cell are uniformly aligned in each layer, showing gradual transition of alignment between cell layers •EC were uniformly distributed in the gel, exhibiting network formation	Wu et al. (2017)
2017	SWCNTs	Various	Various, including the electrospun fibers	Cardiac constructs	Carbon nanomaterials • Mimic the sub-micron scale architecture of the fibrous network of the natural ECM • Mimic the anisotropic alignment of components in the myocardial ECM • Improve the electrical signal transfer within the scaffold	Various cardiac cells (mainly cardiomiocytes)	• No	• Aligned fibers = aligned cells •Increased cardiomyocyte adhesion and scaffold conductivity •Increased electrical conductivity •Presence of CNM yields materials that are more capable of accommodating the compressive strain of the heart •100% and 30% in the elastic and compressive moduli, respectively, in CNT-based scaffolds •Improved cellular proliferation and better attachment of cardiomyocytes •Better retention, spreading, adhesion, and viability •CNTs induce clear membrane segregation and the formation of desmosomes •CNTs induce sarcomere organization, increased packing density, and increased syncytia area in cultured cardiomyocytes •Cardiac cells grown on CNTs-enhanced scaffold are more homogenous, intact and locally aligned •Promotion of cardiomyocyte-like morphology in the hMSCs, increased expression of cardiac markers •CNMs upregulate genes crucial for cardiomyocyte contraction and electrical communication between cardiomyocytes	Dozois et al. (2017)
	MWCNTs								
	CNH (carbon nanohorns)								
	GO								
	Rosetta nanotubes								
	Carbon nanofibers								
2017	SWCNTs	GelMa	GelMA/CNTs hydrogels in the form of films	*In vitro* tissue engineering of myocardium	• CNT hydrogels provide topographical, electrical and mechanical cues for proper organization and tissue formation •CNTs support or direct the attachment, electrical coupling and function of CMs	Neonatal rat ventricular myocytes (NRVMs) isolated from 1-day-old Sprague-Dawley (SD) rats	• No	• NRVMs exhibited aligned and elongated morphology with massive actin striation •Significant increase in both sarcomere length and Z-line width •CNTs facilitated cardiomyocyte assembly into an organized and dense tissue, with a strong and anisotropic contraction potential •CNTs promote intercalated disks formation in the ECTs •Cells grown on CNTs materials contracted synchronously at day 7, and showed apparent spontaneous electrical activity in calcium images •CNTs activate the β1-integrin signaling at the cell membrane and trigger the downstream signaling, regulating the formation of gap and mechanical junctions	Sun et al. (2017)
2018	SWCNTs MWCNTs	Various	Various, including the electrospun fibers	Multiple cardiac applications	• CNTs grant materials with electrical conductivity and improve mechanical properties of polymer scaffolds	Various cell types	• No	• Inclusion of single- or multi-walled CNT into various polymeric scaffolds results in significant improvement in electrical stimulation and signal transduction through CM •Greater potential of augmenting CM growth, proliferation, differentiation, maturation and functioning, synchronous beating •Aligned CNT in polymer scaffolds create synthetic microenvironments improving functionality of CM •Better mechanical properties •Improved remodeling of intercalated discs	Gorain et al. (2018)

Overall, scientific data reports that cardiac cells grown on CNT-modified scaffolds exhibit improved proliferation rates, have spontaneous and synchronous beating, have elongated morphology, and synthesize increased levels of differentiation markers, with better results obtained when scaffolds are of fibrous morphology and even furthermore improved when electrical stimulation is applied (Table 8) (Ashtari et al., 2019). These indicate the importance of combining multiple biofunctional cues in the regeneration of cardiac tissue. Most often, the improved performance of cardiac cells grown on CNT modified scaffolds can be attributed to an increased electrical conductivity, changes in nanoroughness and altered mechanical properties. Gorain et al. pointed out that presence of CNTs significantly enhances the scaffolds’ performance: improving both the mechanical and electrical properties, augmenting CM growth, proliferation, differentiation, maturation and functioning, synchronous beating, and enhancing remodeling of intercalated discs (Gorain et al., 2018). It has been pointed out that the addition of CNTs leads to the creation of biomaterial composites with electrical conductivity similar to the native myocardium (i.e., between 0.06 and 0.4 S/m (Miklavčič et al., 2006)). However, it is important to point out that it is not uncommon for scientists to use CNTs as-received, without providing any physicochemical data, and sometimes even lacking information about the manufacturer, or whether the materials used were SWCNTs or MWCNTs. This practice leads to large discrepancies in the obtained data and makes it hard to analyze and compare CNT CVD results. It is our strong belief that further progress in the field of CNT-modified cardiac tissue scaffolds will not be made until one is able to know exactly what kind of material was used, how it was synthesized, and what are its properties, as CNT performance is strongly dependent on its structure, agglomeration state, chemistry, type, and amount of functional groups.

In a different approach, McCauley et al. (McCauley et al., 2019) were able to fabricate a completely new material–a carbon nanotube fiber (CNTf) and used this as sutures for transmitting electrical stimulation between regions of the heart and across epicardial scar in sheep. Without evident cytotoxicity, the material was able to restore the beating function of the heart. This solution was suggested to provide long-term restorative strategies to pathologies that interrupt the efficient electrical transduction in electrically excitable tissue, including the heart.

##### 8.2.4.3 Graphene

The successful fabrication of a single-layer graphene in 2004 by Konstantin Novoselov, Andre Geim and their team (Novoselov et al., 2004) opened up the gate to a plethora of possible applications in various fields of science and industry, including biomedical applications. Significant scientific interest stems from the material’s outstanding properties–most notably, a single layer of graphene is a 2D material, with extremely high mechanical and electrical properties. Traditionally, graphene can be obtained as a deposited layer during chemical vapor deposition, chemical decomposition or direct chemical synthesis processes, or in the form of free-standing flakes by mechanical or chemical exfoliation. More recent methods report unzipping of CNTs to form so-called graphene nanoribbons, or a microwave synthesis (Bhuyan et al., 2016). The biggest issue in working with graphene is its high tendency to agglomerate via pi-stacking which spontaneously recreates the structure of graphite. Thus, instead of working with single-layered graphene, one is in fact using a few-layers of graphite. To circumvent this issue, various techniques reducing the pi-pi attraction by introducing additional repulsive forces are employed, most notably chemical oxidation (Johnson et al., 2015). Indeed, most commercially available graphene is in fact, graphene oxide (GO), obtained by chemical exfoliation of graphite oxide, with functional groups evading re-organization into graphite.

Most scientists work with the as-obtained materials, pointing out better dispersability as factors enhancing graphene biocompatibility (Table 9). However, the electrical conductivity of GO may be insufficient for some applications and thus, some researchers decided to reduce GO to obtain reduced GO (rGO), indicating that this type of material should possess better electrical properties, and its physicochemical properties may be tuned to meet specific cell type requirements (Shin et al., 2016). In this regard, an interesting approach was used by Jo et al., who was able to harvest the positive effect of GO’s functional groups on nanoparticle dispersion, while fabricating a material of fairly good electrical conductivity (still lower than that of the native myocardium, 0.014 S/m compared to between 0.06 and 0.4 S/m) (Miklavčič et al., 2006; Jo et al., 2017). He did it by first preparing the polyacrylamide/GO composites and then mildly reducing them in ascorbic acid, which caused the transition of GO to rGO without compromising the material’s integrity and mechanical properties. Probably, two of the most important studies completed on the application of GO in cardiac tissue engineering include those by Zhao et al. (Zhao et al., 2018) and Bao et al. (Bao et al., 2017). The first indicates the importance of combining multiple biofunctional cues–fibrous morphology, electrical conductivity (obtained by covering the fibers with a graphene nanolayer), and electrical stimulation to enhance the adhesion, proliferation, differentiation, and maturation of stem cell-derived cardiomyocytes. Even though the fabrication of the GO layers could still be improved (specifically, improving the layer’s adhesion), the obtained results are truly remarkable with a significantly up-regulated expression of cardiac markers. Meanwhile, Bao’s study is one of the rare ones to report *in vivo* results, indicating that GO modified hydrogels were able to rebuild cardiac function, restoring functionality close to a healthy heart, as it was before the myocardial infraction (Bao et al., 2017).

**TABLE 9:** Selection of GO and rGO used in cardiac and muscle tissue engineering.

Year	Type of additive	Polymer used	Morphology of matrix	Application	Rationale for this composition	Cells used	ES	Results	Citiation
2016	rGO GO	GelMA	Hydrogel film	Myocardial infraction	• Natural polymers—better biocompatibility •Additive—improved mechanical and electrical properties •rGO better biocompatibility than graphene and GO	NIH-3T3 and cardiomyocytes isolated from 2-day-old Sprague–Dawley rats	Yes, 8116A, Pulse/Function Generator 50 MHz, Hewlett-Packard) produced biphasic waveforms with 50 ms pulses of 3–6 V/cm at 0.5, 1, 2 and 3 Hz.	• Resistance of gel: 4 kΩ	Shin et al. (2016)
								• No cytotoxic effect, better cellular adhesion and spreading with rGO	
								• Cardiac tissues on rGO-GelMA seemed more organized with enhanced cell–cell coupling	
								• Higher spontaneous beating rate on rGO scaffolds	
								• Tissue constructs containing rGO sheets responded to an externally applied electrical field with synchronous contraction	
								• Constructs made from rGO exhibited better cardiac function than the ones with GO	
2017	GO	Chitosan	Injectable hydrogel	Myocardial infraction	• Injectable—facile administration •Self-healing—can recover upon deformation caused by heart •Mussel inspired protein (polydopamine)—self-adhesion, reduction and dispersion of nanoparticles (GO)	HEF1 fibroblast differentiated from the human embryonic stem cell line WA09 Cardiomyocytes derived from human embryonic stem cell line (hESC) Culture with extracts	No	• Electrical conductivity: 0.122 S/m	Jing et al. (2017)
								• The material is “self-healing”	
								• Faster cross-linking with GO present	
								• GO loaded material was more adhesive than non-loaded up to concentration of 0.5 mg/ml	
								• GO content lower than 0.75 mg/ml was beneficial for cell proliferation and viability and increased beating rate	
2017	GO reduced to rGO (0.3%)	Polyacrylamide (PAAm)	Hydrogel films	Tissue engineering	• Hydrogel to mimic the mechanical properties of the native soft tissues and study cellular behavior	C2C12 myoblasts	Yes, after reaching confluence, 1 V/cm, 10 ms duration, 1 Hz, 4 h for 3 and 7 days	• Conductivity 0.014 S/m	Jo et al. (2017)
					• Conductive additive for the biomaterial to deliver multiple critical signals to cells/tissues			• rGO enabled cellular adhesion and proliferation	
								• Materials support myogenic differentiation and fusion	
								• ES upregulated expression of myogenic genes	
2018	rGO film	Silk fibroin	Electrospun fibers—aligned and random, covered with GO film	Tissue engineering *in vitro*	• To have electrically conductive scaffolds without the need to reach percolation threshold in composites	Cardiomyocytes from neonatal rats	Yes, starting at 3rd day of culture, ES for 4 days (1 Hz, 5 V/cm^−1^)	• Resistance measured by 4-point probe reduces linearly with increasing thickness of the layer	Zhao et al. (2018)
								• rGO is biocompatible and improves striation and maturation (more pronounced on thicker layers, indicating a critical role of topological cues in regulating cardiomyocyte morphology)	
								• Expression of α-actinin is significantly promoted by a thinner rGO layer	
								• rGO layer enhanced expression of Cx-43—enhanced enhance cell–cell communication	
								• Nanofiber alignment is more effective for the organization of proteins than gene transcription and protein expression	
								• Electrical stimulation significantly increases the expression of cTnI and Cx-43 on the aligned rGO/silk	
								• Coupling between the direction of electrical stimulation and the tissue orientation is essential for the enhancement of cell–cell coupling	
								• Beating rate and intensity of cardiac tissues on rGO/silk scaffolds are significantly higher than on pure silk and this effect can be improved with electrical stimulation	
								• The cardiac tissues in the aligned silk group exhibited the weakest cell–cell communication	

## 9 Designing mechanical properties

Stem cell differentiation and cardiomyocyte beating behavior are known to be sensitive to scaffold rigidity and elasticity. In their research article from 2008 (Engler et al., 2008), Engler et al. proved that cardiomyocyte beating is inhibited if the matrix is too stiff (>50 kPa, stiffness of scar tissue), because cells are forced to perform work that exceeds their native limit. Meanwhile, if the scaffold is too soft (<1 kPa), the cells preserve their beating behavior, but do less work than they should in a healthy tissue. As Chopra et al. suggested, this may lead to the formation of disorganized actin networks and sarcomeres, causing a decrease in tissue functionality (Chopra et al., 2012). It is hypothesized that the optimal stiffness of the scaffold for cardiac tissue engineering is between 10 kPa–50 Pa, which is close to that of the healthy tissue. In these conditions, cardiomyocytes are able to form well organized sarcomeres, exhibiting actomyosin striation and 1-Hz beating (Engler et al., 2008; Chopra et al., 2012). Again, this stresses the importance of biomimetism in designing a material that can be used to successfully regenerate the damaged heart. Designing scaffold stiffness for steering cellular behavior is the subject of recent review article by Rajendran et al. (Rajendran et al., 2023), as well as the studies by Zhong et al. (Zhong et al., 2020) and Irawan et al. (Irawan et al., 2018). The readers are kindly referred to these excellent articles if more details on how the substrate’s mechanical properties affect cellular functions are needed.

In a 2012 review by Sheehy et al., the authors list the importance of mechanical cues in dictating stem cell fate during early development of the heart. It can, thus, be postulated that by mimicking mechanical properties of the heart ECM in the early development stages of heart tissue formation, tissue engineering scaffolds can induce the differentiation and maturation of cells, guiding them along the cardiac lineage. This has been found to be true for embryonic cardiomyocytes (Sheehy et al., 2012). Similar are the conclusions of a 2014 study by Hazeltine et al., reporting that substrate stiffness is the determinant in the early stage of human pluripotent stem cell differentiation, pushing the differentiation trajectory towards mesendoderm. The two most favorable stiffness values were 50 and 20 kPa. Interestingly, at later stages of cellular differentiation, this impact was found to be less pronounced (Hazeltine et al., 2014).

Most biomimetic biopolymers and hydrogels are too soft to fully mimic the mechanical properties of the native myocardium. To aid this issue, various approaches have been undertaken. One idea concerns preparing blends with other stiffer polymers, and this subject has already been raised in Sections 3.1 and 5.1 of this review article. Preferably, a mixture of conductive and non-conductive polymers could be obtained to render a final product more mechanically suitable and electrically conductive. The introduction of various additives (micro and nano) has also been found to improve the polymers’ mechanical properties, as already raised in Section 5 of this review, in particular for CNTs (Sirivisoot and Harrison, 2011; Ostrovidov et al., 2014; Ahadian et al., 2016; Ahadian et al., 2017; Dozois et al., 2017; Gorain et al., 2018) and GNPs (Li et al., 2016; Navaei et al., 2016). In all of these materials, the presence of the additives resulted in obtaining electrically conductive and mechanically biomimetic scaffolds, which significantly enhanced cell maturation. Using modifications that have such a dual function is certainly the most favorable option.

A third strategy to improve the material’s mechanical properties is to induce chemical changes to its structure, which is typically done by crosslinking. This has been employed in various studies, for example by Fu et al. in PDMS substrates (Fu et al., 2017), by Choi et al. on gelatin-PEG-tyramine co-polymers (Choi et al., 2017), or by Shi et al. in hyaluronic acid-based hydrogels (Shi et al., 2023). It is worth noting that the latter study employed the use of numerous modifications to provide the composite with various biofunctional properties: CNTs to enhance the electrical conductivity (sadly, the reported CNTs were of unknown origin and their final concentration in the polymer was not defined in the report) and mixing of biopolymers with synthetic polymers to further improve the mechanical properties.

In our opinion, the most optimal solution for culturing stem cell-derived CMs would be to use a scaffold with steerable or adaptable mechanical properties. At first, it should mimic the stiffness of the developing cardiac tissue (4–11 kPa), but should then become stiffer and stiffer, training the cells and forcing them to perform an increasing amount work, until it reached the limit of that in the adult native tissue. By this means, mechanical properties of the scaffold can induce and maintain the progressing cellular maturation, which is guided by the cellular contractile work (Ulmer et al., 2018).

There is an analogy between electrical conductivity of the scaffold and the benefits of electrical stimulation with mechanical properties of the substrate (most importantly, stiffness), and use of mechanical stimulation. The scaffold should be designed so as to mimic the natural ECM, i.e. the scaffold should be able to transmit electrical signals from cell to cell, and be able to support them mechanically. At the same time, exogenous impulses, such as electrostimulation and mechanostimulation, may be used to mimic the natural occurrences in the living organism, with ECM fibers moving and contracting, and electrical fields naturally present *in vivo.* Such stimuli had been proven to enhance cellular adhesion, proliferation, differentiation and maturation (Godier-Furnémont et al., 2015). Further benefits of using electrostimulation and mechanostimulation can be found in a review by W. L. Stoppel, D. L. Kaplan and L. D. Black III (Stoppel et al., 2016).

## 10 Electrostimulation (ES) and mechanostimulation (MS)

For the *in vitro* tissue engineering approach, ES using a carefully selected stimulation regime (time, amplitude, frequency, stimulation onset in the cell culture, etc.), has proven to enhance cellular adhesion, guide migration, enhance viability and proliferation, and provide differentiation cues for stem cells, by affecting various signaling pathways (Mooney et al., 2012; Kwon et al., 2016; Thrivikraman et al., 2016; Li et al., 2017b; Thrivikraman et al., 2018) (Table 10). In *in vitro* studies, external ES is aimed to mimic endogenous electrical fields that naturally occur *in vivo* and are responsible for multiple phenomena including tissue regeneration. Considering mimicking the maturation of the heart *in vitro*, ES at first can be used to alter the cellular metabolism and then, mimic the signals from the pacemaker cells. By mimicking the signal from the pacemaker cells, exogenous ES can alter the cellular physiology, forcing them to switch from spontaneous, unsynchronized pacing, to synchronized pacing triggered only by external factors. In their extensive review from 2018, Thrivikraman, Boda and Basu identified the major effects of ES stimulation on stem cells, suggesting different mechanisms of interactions: alteration of biochemical signaling pathways, calcium diffusion transients, induced cytoskeletal reorganization, ATP synthesis, reactive oxygen species generation and synthesis of heat shock proteins (Thrivikraman et al., 2018).

**TABLE 10:** The influence of electrical stimulation on cellular proliferation, maturation and differentiation along the cardiac cell lineage from different stem cells including mesenchymal stem cells.

Cell type	Conductive nanoadditive	Stimulation regime	Results	Ref.
hPSCs	-	• 1 week of AC ES of progressing frequency (from 1 to 6 Hz)	• Enhanced maturation	Nunes et al. (2013)
iPS(Foreskin)-2	-	• 5th day of culture • Square wave • 2 V/cm • 1 Hz • 1 ms pulse width • 5 min of stimulation	• Enhanced beating, increased expression of differentiation markers	Hernandez et al. (2016)
hCPCs	-	• 1st day of culture • Square wave • 3.4 V/cm (biphasic and monophasic) • 1 Hz • 2 ms pulse width • 3 days of constant stimulation	• Slowed down proliferation rate	Pietronave et al. (2014)
			• Spindle-like morphology	
			• Improved differentiation and maturation	
MSCs	+, CNTs	• After reaching confluency • Square wave • 0.15 V/cm • 1 Hz • 2 ms pulse width • 14 days	• Spindle-like morphology	Mooney et al. (2012)
			• CNTs in the presence of ES provide biomimetic stimulus for differentiation, maturation and improved cell to cell cross-talk	
hMSCs	+, GNPs	• 3rd day of culture • Square wave • 0.1 V/cm • 1 Hz • Duty cycle of 10% • 14 days, 15 min/day	• ES + GNPs guide differentiation along cardiac lineage, irrespective of surface chemistry and morphology	Thrivikraman et al. (2016).
iPSCs		• After 7 days of compaction in cardiac tissue–formation platform, begin ES • DAY 1: 2-ms monophasic square repeating pulses, delivered at 2 Hz and an amplitude of 4.5 V/cm • DAYS 1–12: Increase the stimulation frequency daily by 0.33 Hz (from 2 Hz at day 0 of stimulation to 6 Hz on day 12 • DAYS 12–15. Keep the stimulation at 6 Hz • DAYS: 15–21. Drop the stimulation to 2 Hz	• Generation of cardiac tissue with mechanical and electrophysiological properties close to those seen in mature myocardium	Ronaldson-Bouchard et al. (2018)
Cardiomyocytes from rat embryo heart		• Trains of bipolar electrical pulses (0.1 mA, 1 ms, 1 Hz) applied for 30 min each day for six continuous days	• Surface coverage of α-actinin, cTnT, and Cx43 is significantly enhanced	Zhang et al. (2018)

In the heart, it is anticipated that direct current (DC) stimulation is able to mimic the electrical signals found in early tissue development stage, affecting cellular migration and morphology, while an attenuated current (AC) signal can mirror the reactions occurring in the adult heart, dictating the fate of the stem cell niche, affecting cardiomyocyte beating behavior, influencing assembly of sarcomeres and myotubes. Thus, in planning ES experiments and selecting a suitable stimulation regime, cell type and desired outcomes should be carefully considered. Currently, a majority of the studies report AC stimulation, most often, square-wave, with a frequency of 1–2 Hz, pulse duration of 1–2 ms and amplitude between 0.1–10 V/cm (Tandon et al., 2009; Thrivikraman et al., 2018). Nunes found that 1 week of ES stimulation of progressing frequency (from 1 to 6 Hz) is able to enhance cardiomyocyte differentiation that were different from human pluripotent stem cells (hPSCs) and cultured in a specially designed 3D construct, Biowire (Nunes et al., 2013). Hernandez and his team reported that cardiomyocytes differentiated from induced pluripotent stem cells (iPS(Foreskin)-2) as demonstrated by enhanced beating, increased expression of differentiation markers, etc. under AC ES (Hernandez et al., 2016). Meanwhile, Pietronave and his team researched human cardiac progenitor cells (hCPCs) and studied the impact of both biphasic and monophasic AC ES on cellular morphology, proliferation, viability and differentiation. While ES did not affect cellular viability, proliferation rate slowed down, which was accompanied with adopting of spindle-like morphology, improved differentiation and maturation (Pietronave et al., 2014). Thus, AC ES was found to improve differentiation and maturation efficiency of cardiomyocyte progenitor cells from different sources.

The synergistic effect of conductive nanoparticles and ES were studied with very promising results. In 2012, Mooney and co-workers studied effect of the presence of COOH–functionalized SWCNTs, both in the medium and in a PLA fibrous scaffold, on the differentiation and maturation of ES-stimulated mesenchymal stem cells (MSC). She had found that electrical stimulation alone was able to force cellular alignment, with a more pronounced effect observed in the presence of CNTs. Furthermore, the CNTs were found to provide a biomimetic stimulus for MSC differentiation, accelerate their maturation and improve cell to cell cross-talk (Mooney et al., 2012). Similar results were also reported by Thrivikraman and his co-workers, who worked with GNPs. The team found that AC electrical stimulation combined with GNPs internalized by cells was able to guide the differentiation of human mesenchymal stem cells (hMSCs) along a cardiac-like lineage without the use of any exogenous growth factor even on the surface of a glass coverslip (Thrivikraman et al., 2016). Because in these studies mesenchymal cells are used instead of actual stem cells, the obtained results should be treated as a guidance for cell culture conditions that may be favorable for cardiac-like cells. These studies point out the beneficial impact of synergy between conductive nanoparticles and electrical stimulation in promoting cellular differentiation into the cardiac lineage.

Some advantages of employing MS can be found in a recent study by Zhao et al. (2023). The benefits of combining MS with ES in *vitro* cultures of cardiac cells were analyzed in-depth in the 2016 review by Stoppel et al. (2016) and the readers are kindly referred to this excellent study for more details on the rationale, benefits and possible ways to employ ES and MS, both separately and in combination. It is worth noting that combining ES and MS is justified from the biological point of view as it mimics the natural environment of cardiac cells. Various approaches can be taken to induce both stimulants, both separately and concurrently, as presented in the two aforementioned reviews. One possibility, as suggested by Gouveia et al., is the use of piezoelectric materials that would spontaneously generate ES while under mechanical stress and *vice versa*. This simple idea yielded some promising results: cell attachment and alignment promoted, ratio of cell populations maintained, cell-cell communication and metabolic maturation enhanced, and cardiomyocyte (CM) contractility maintained for at least 12 days. Sadly, the study did not investigate whether or not such a system could induce or enhance the differentiation and/or maturation of juvenile cells and instead focused on maintaining the viability of mature cardiomyocytes. Hence, further studies are needed to verify any possible positive outcomes in these applications (Gouveia et al., 2017).

**FIGURE 10:**
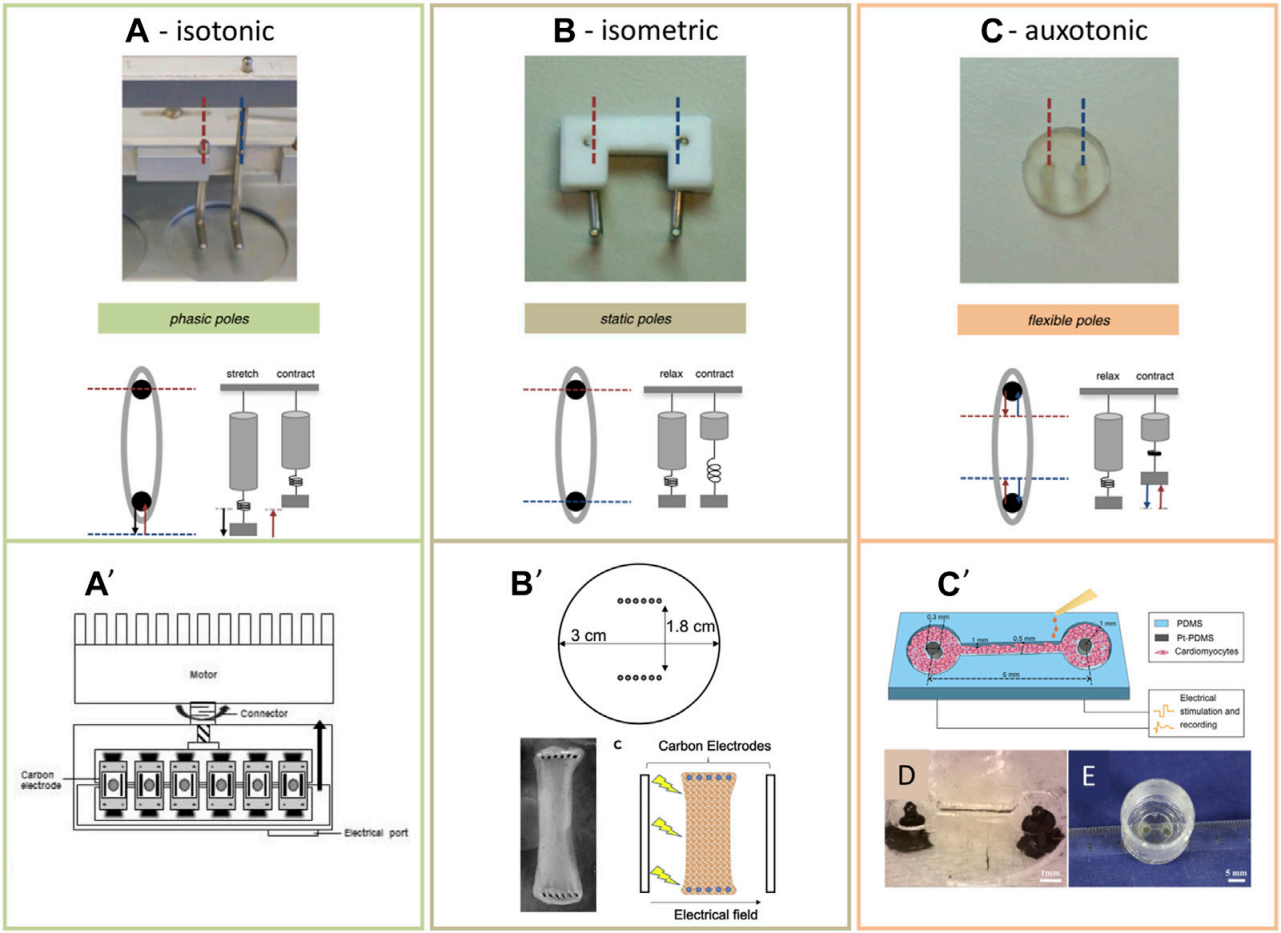
**(A–C)** different modes of mechanical stimulation, as presented by Liaw and Zimmermann (Liaw and Zimmermann, 2016), combined with their practical realizations, wherein MS is combined with ES: **(A′)**: C-stretch system from IonOptix, as presented by Kroll et al. (Kroll et al., 2017); **(B′)**: a layer-by-layer assembly of cells placed on top of two arrays of stiff needles. ES was employed by immersing two counter electrodes, perpendicular to the immersing two counter electrodes, perpendicular to the needle axes (C-pace system) (Pretorius et al., 2022); **(C’—E)**: the cells are seeded inside the specifically shaped channels, which force their alignment around the elastic electrodes that can be used for electrical stimulation (Zhang et al., 2018). **(A–C)**: N.Y. Liaw, W.-H. Zimmermann, Mechanical stimulation in the engineering of heart muscle, Advanced Drug Delivery Reviews 96 (2016) 156–160 https://doi.org/10.1016/j.addr.2015.09.001 with permission from Elsevier, Copyright 2016. **(A′)** Reprinted from Kroll et al. (2017) with permission from Elsevier, Copyright 2017. **(B′)** Reprinted from Pretorius et al. (2022), with permission form Elsevier, Copyright 2022 (CC BY-NC-ND). **(C’—E)** Reprinted from Zhang et al. (2018) with permission from Elsevier, Copyright 2018.

Apart from using the piezoelectric materials, methods of delivering MS into a system can be grouped into 3 major categories (Liaw and Zimmermann, 2016), as seen in Figure 10. The first one, also referred to as isotonic contractions, is achieved by physically stretching the cell culture. This can be done either by stretching the entire cell well (or only its bottom), or by seeding the cells on or around pillars which then can be moved inside the culture. Stretching of the cell well as a whole is employed in a commercially available C-Stretch system from IonOptix, wherein displacement of the cell well holders can cause cyclic stretch. The same idea can also be achieved by stretching of the elastic cell well bottom by changing pressure in the gas or liquid filled chambers located below it (Cortes et al., 2020; Correia Carreira et al., 2021; Zhao et al., 2023). Alternatively, specially designed poles can be placed inside the cell wells or at its bottom and the moved by some sort of physical force–mechanic or magnetic (Zhao et al., 2023).

The second and third methods of introducing MS operate without any exogenous force that introduces the stretch and instead, the cells and the compacted scaffolds or ECM themselves are the source of the strain. These systems, referred to as isotonic and auxotonic contractions by Liaw and Zimmermann (Figure 10) (Liaw and Zimmermann, 2016), require arranging the cell and scaffolds into compact and organized tissues around specifically designed poles. When such poles are made of stiff materials, the arrangement and densification of the matrix introduces isometric strain into the culture. However, when such poles are fabricated from elastic materials, such as polydimethylsiloxane (PDMS), arrangement of a dense, tissue-like structure, combined with its self-pacing, introduce auxotonic contractions into the system. This is a very smart and elegant approach in which the cells generate the MS by themselves.

An example of employing the isotonic stretch along with ES can be found in a study by Pretorius et al. (Pretorius et al., 2022). A 2.1 mm thick cell sheet, fabricated by assembling 3 consecutive layers of juvenile cardiomyocytes inside the fibrin hydrogels, was stretched by being pierced between 2 sets of surgical needles (each composed of 6 needles, set 1 mm apart). The two sets created parallel lines, facing each other at a 18 mm distance. ES of two amplitudes (namely, 15 V and 22 V) was then employed (2 ms pulses at a 2 Hz frequency) perpendicularly to the needles, by using the C-pace system. It was observed that double stimulation (ES + MS) significantly enhances the maturation of the iPSC–derived CMs, which was further boosted by using a higher amplitude of voltage. After 10 days of culture, the CMs in this group had sarcomeres of physiologically relevant lengths (above 1.9 µm), with biochemical maturation markers significantly elevated. The authors suggested that positive outcomes could be further boosted by using a co-culture with cardiac fibroblasts, which we find probable. In our opinion, the observed effects could also be improved if the ES and MS were not perpendicular to one another and parallel instead. Moreover, we found that study lacks an important experimental detail, namely, in the ES protocol, the length of stimulation is not provided. Further, environmentally-friendly materials should be used.

Auxotonic systems are probably amongst the most popular solutions used to combine ES with MS. Some examples of these can be found in the studies done by the teams of Vunjak-Novakovic and Radisic (Nunes et al., 2013; Ronaldson-Bouchard et al., 2018; Ronaldson-Bouchard et al., 2019; Zhao et al., 2019), wherein protocols for enhancing the maturation of cardiomyocytes are developed. One version of the system, Biowire II, progressed into industry (originally as TARA biosystems, currently as a part of Valo Health) offering the first-of-its-kind cardiovascular screening platform (Nunes et al., 2013; Zhao et al., 2019). In what seems to be a new approach to the same problem (Ronaldson-Bouchard et al., 2018; Ronaldson-Bouchard et al., 2019), two elastic poles were used to induce auxotonic stretch to a CM–fibroblast co-culture in a fibrin hydrogel, and ES was induced parallel to the MS. The ES was conducted in the mode of “intensity training” (2 weeks at a frequency increasing from 2 Hz to 6 Hz by 0.33 Hz per day, followed by 1 week at 2 Hz, with voltage set to 4.5 V/cm). As a result, the speed of cellular maturation was enhanced three-fold, with sarcomere lengths exceeding 2.2 µm, the density of mitochondria was improved by 30%, and many important biochemical markers were also significantly elevated. These important studies suggest the need of employing a combinatorial approach which involves the use of different stimulants, biomaterials, and co-cultures in obtaining mature tissue fragments *in vitro.* Apart from the simplicity of using the auxotonic systems in introducing mechanical stretch, another advantage lies in the fact that the displacement of elastic poles can be used to monitor the pacing of the maturating cultures, as demonstrated in the studies by Legant et al. (Legant et al., 2009) and by Ma et al. (Ma et al., 2019). The simplicity and versatility of this strategy contributes to the fact that right now, auxotonic systems are the go-to methods for fabricating maturated tissue constructs out of iPSCs-derived cardiomyocytes.

A combination of auxotonic and isotonic stress applied to the system can be found in a study by Leonard et al. (Leonard et al., 2018). In what the authors refer to as the afterload simulator, a tissue construct made of iPSC-CMs embedded inside the fibrin gel was compacted around two posts. One post was made out of a stiff material, while the other had a varied elasticity. Pacing of the culture with ES would cause tissue contraction and displacement of the post which then resisted tissue relaxation–thus creating an “afterload” condition. It is worth noting that the authors tested different stiffnesses of their system to verify the optimal afterload for CM maturation. It was found that the contractile function was enhanced at moderate (0.45 μN/μm) afterload values and plateaued at higher values (1.2 μN/μm and 9.2 μN/μm). The structural maturation of cells improved on all of the “stressed” cultures, and to a similar extent regardless of the post stiffness. At the same time, markers of functional (calcium transient kinetic) and biochemical cellular maturation (increased expression of mature CM genes) were enhanced as a function of increasing afterload, but the same was also observed for pathological hypertrophy markers. Hence, it was suggested that an optimal value of the afterload (1.2 μN/μm) exists and that it can be used to speed up CM maturation.

## 11 Summary

Like any other tissue of a living organism, cardiac tissue is a complex structure, stemming from the different functions it performs. Unlike any other tissue, it has almost zero regenerative potential, due to the absence of the stem cell niche. This inability to self-regenerate, combined with frequency of CVDs, and absolute essentiality of this organ, create an absolute necessity to study the development of pathogenic diseases and engineer novel regeneration strategies. Even though it is now relatively facile to obtain cardiomyocytes from patient-specific iPSCs, there is still a long way to travel before these can be translated to medicine. The most important hurdle is an inability to create tissues of a mature phenotype *in vitro,* and in a relatively short time–i.e., without consuming excessive resources or increasing the risk of failure.

The current socioeconomic and ecological impact of CVDs stems from two factors: first is their prevalence in the modern society and second is the overall cost of research involving their pathogenesis and regeneration. Numerous strategies suggest creating mature tissue constructs *in vitro,* which could be used in treating damaged tissue *in vivo,* in studying the development of various diseases and their treatment, or in evaluating the cardiotoxicity of drugs. Such strategies could successfully reduce the current carbon footprint associated with treating CVDs. Moreover, we need to take a fresh look at the materials used for treating CVD as many involve polymers and/or other materials which use environmentally toxic agents. *In vitro* and *in vivo* testing also involves the use of tremendous amounts of polymers which are not environmentally-friendly. Designing solutions that would reduce the time needed for cell culture to develop a mature phenotype could significantly reduce the amount of resources and consumables–this is important, because current procedures often require culturing the cells for more than 1 year. Even though significant progress has been made on this subject in the recent years, a perfect solution has yet to be developed. Emphasis must be placed on using new, green-derived, sustainable, and biodegradable materials not just for CVD but across all of biomaterials. These include animal, plant and microorganism-derived materials, most notably, collagen.

Knowledge about the complexity of cardiac tissue, various interactions between different cell types, and between cells and the cardiac tissue ECM, point out directions in which the field should be evolving. The combination of biomimetic scaffolds with biological and physical cues has already yielded impressive results. For example, collagen scaffolds were successfully used for co-culturing CMs with fibroblasts, while introducing exogenous electrical stimulation (e.g. (Ronaldson-Bouchard et al., 2018; Ronaldson-Bouchard et al., 2019; Zhao et al., 2019)). Still, no single solution exists to mimic all the interactions from within living tissues. It can be suggested that a combination of a majority of factors is the only route to obtaining fully mature cardiac tissues *in vitro,* which could then be used for drug screening, various pathogeneses, and cardiac regeneration *in vivo.* Among different possibilities, we possibilities, we believe following approaches are the most promising to both fabricate improved CVD biomaterials and save the environment:

1) Use scaffolds that are biomimetic to the native cardiac tissue ECM (both in their chemistry, electrical and mechanical properties and morphology), preferably, fibrous scaffolds made of collagen type I, perhaps mixed in-mass, or surface-modified with laminin, elastin or fibrin;
2) In-mass modification with various additives that alter the scaffold’s mechanical and electrical properties, thus making it mimetic to the native cardiac tissue ECM. In our opinion, fibrous morphology and benign chemistry of environmentally-friendly carbon nanotubes make them perfect candidates in this matter. Furthermore, when these are short and highly functionalized, high biodegradability can be achieved;
3) In-mass modification of scaffolds with bioactive molecules for their controllable release. The most promising ones include: albumin-modified fatty acids (which could force a switch in the CM metabolic pathway), triiodothyronine (inducing a switch from fetal N2BA to an adult N2B titin isoform) and dexamethasone (both of which affect the CM electrophysiology, and IGF-1 (to induce the hyperplastic growth)) (Martyniak et al., 2023). Preferably, these should be released in a switchable manner, so as to mimic their natural occurrence within the developing and maturing heart;
4) A co-culture with cardiac fibroblasts which could aid in scaffold remodeling and secrete specific signaling molecules that further drive the CM maturation;
5) Introducing exogenous electrical and mechanical stimulation which could “train” the cardiac tissue, thus forcing CM maturation; and
6) Finally, in our opinion, CM culture should be dynamic–the mechanical and electrical properties of the growing tissue should be controllable. Tricking the cells that their surroundings are becoming mature is a known driving force of inducing faster cellular maturation.
